# Incorporating historical controls in clinical trials with longitudinal outcomes using the modified power prior

**DOI:** 10.1002/pst.2195

**Published:** 2022-02-06

**Authors:** Hongchao Qi, Dimitris Rizopoulos, Emmanuel Lesaffre, Joost van Rosmalen

**Affiliations:** ^1^ Department of Biostatistics Erasmus University Medical Center Rotterdam The Netherlands; ^2^ Department of Epidemiology Erasmus University Medical Center Rotterdam The Netherlands; ^3^ I‐Biostat KU‐Leuven Leuven Belgium

**Keywords:** Bayesian statistics, clinical trials, historical borrowing, informative prior, modified power prior

## Abstract

Several dynamic borrowing methods, such as the modified power prior (MPP), the commensurate prior, have been proposed to increase statistical power and reduce the required sample size in clinical trials where comparable historical controls are available. Most methods have focused on cross‐sectional endpoints, and appropriate methodology for longitudinal outcomes is lacking. In this study, we extend the MPP to the linear mixed model (LMM). An important question is whether the MPP should use the conditional version of the LMM (given the random effects) or the marginal version (averaged over the distribution of the random effects), which we refer to as the conditional MPP and the marginal MPP, respectively. We evaluated the MPP for one historical control arm via a simulation study and an analysis of the data of Alzheimer's Disease Cooperative Study (ADCS) with the commensurate prior as the comparator. The conditional MPP led to inflated type I error rate when there existed moderate or high between‐study heterogeneity. The marginal MPP and the commensurate prior yielded a power gain (3.6%–10.4% vs. 0.6%–4.6%) with the type I error rates close to 5% (5.2%–6.2% vs. 3.8%–6.2%) when the between‐study heterogeneity is not excessively high. For the ADCS data, all the borrowing methods improved the precision of estimates and provided the same clinical conclusions. The marginal MPP and the commensurate prior are useful for borrowing historical controls in longitudinal data analysis, while the conditional MPP is not recommended due to inflated type I error rates.

## 1 INTRODUCTION

Longitudinal studies are common in clinical settings where health‐related outcomes are repeatedly measured over time. Randomized clinical trials often have a longitudinal nature in the sense that outcomes are measured before and after the intervention. The primary analysis of clinical trials is typically based on a single clinical endpoint, but the analysis of longitudinal outcomes may yield more precise estimates and statistical power.

When analyzing the current clinical trial, it may be sensible to borrow information from similar historical clinical trials to gain more statistical power without increasing the required sample size. Fortunately, information from previous studies is often available in clinical trials. The reason is that clinical trials focusing on the same disease may have a variety of target treatments, but the control arms are often similar to each other.^1^ It is appealing to borrow information from a historical control arm, which may allow researchers to save trial resources on the current control arm and obtain more accurate estimates, increased statistical power and reduced type I error rate.^2^ However, it is obvious that simply pooling the current study and the historical control arm is not appropriate. Several statistical methods have been proposed to downweight information from the historical control arm, including the power prior, the commensurate prior, and the meta‐analytic‐predictive (MAP) method.^3^, ^4^, ^5^

The power prior proposed by Ibrahim and Chen raises the likelihood of historical data (historical likelihood) to a specific power α to generate a downweighted prior with historical information^6^. There are two versions of the power prior. The first type is to fix the power parameter in advance,^3^, ^7^ and the other is to estimate the power parameter based on observed data.^6^, ^8^, ^9^ The modified power prior (MPP) method discussed in this article belongs to the second category, where the power parameter can be estimated in a fully Bayesian way.^9^ In the commensurate prior method, the parameters of the current study have a distribution centered on the corresponding historical parameters.^4^ For the MAP, the parameters from the historical and current study are assumed to be exchangeable, that is, they originate from the same distribution.^5^

The MPP has been implemented for univariate (binary, Gaussian, survival) endpoints,^8^, ^9^, ^10^, ^11^ but the developments for longitudinal endpoints are quite limited. Neelon et al.^7^ have applied the power prior with a prespecified power parameter in a longitudinal pediatric study. However, it is unclear how to specify the amount of historical information to be borrowed before knowing the level of compatibility between the historical data and the current data. The MPP method, which is a dynamic borrowing method that takes the observed data into account when determining the amount of historical borrowing, appears to be more reasonable.

The objective of this article is to extend the MPP to the analysis of longitudinal studies, such as RCTs, and to evaluate its performance in this setting via a simulation study and a real‐life data analysis study. The MPP approach will be illustrated using the data from the Alzheimer's Disease Cooperative Study (ADCS), a large‐scale Alzheimer's disease research network based in the United States.

The article develops as follows. Section 2 introduces the linear mixed model for longitudinal studies, with a focus on RCTs. Section 3 provides an overview of Bayesian borrowing methods. Section 4 describes the implementation of the MPP method in a longitudinal data analysis. Section 5 presents the implementation of an alternative method in longitudinal data analysis. Section 6 discusses the design and the results of the simulation study. Section 7 illustrates the implementation of the MPP in the analysis of the ADCS data. In the final section, we further discuss our findings and provide some general conclusions.

## 2 THE LINEAR MIXED MODEL IN CLINICAL TRIALS

Consider a longitudinal study with Gaussian responses based on n subjects, where the ith subject $\left(i=1,2,\ldots ,n\right)$ has $m_{i}$ repeated measurements. A linear mixed model (LMM) to fit such data can be formulated as:

(1) $$y_{i}=X_{i}\beta +Z_{i}b_{i}+\varepsilon_{i},$$

where $y_{i}$ is the $m_{i}\times 1$ vector of responses of the ith subject, $X_{i}$ is a $m_{i}\times p$ design matrix of fixed effects for the ith subject where p is the number of fixed effects, β is a p×1 vector of fixed effects. $Z_{i}$ denotes a $m_{i}\times q$ design matrix of random effects for the ith subject, q is the number of random effects, $b_{i}$ denotes a q×1 vector of random effects for the ith subject and $b_{i}∼N\left(0,G\right)$ where G is a q×q covariance matrix of the random effects. $\varepsilon_{i}$ is the random error for the ith subject, $\varepsilon_{i}∼N\left(0,R_{i}\right)$, and $R_{i}$ often has the form $\sigma^{2}I_{m_{i}}$.

In an RCT, patients are randomized at baseline, and hence at baseline there is no difference in mean response between the treatments. Often we assume a linear evolution over time in which case the LMM takes the form:

(2) $$y_{\mathrm{it}}=\beta_{0}+\beta_{1}\times {\text{time}}_{\mathrm{it}}+\beta_{2}\times {\mathrm{trt}}_{i}\times {\text{time}}_{\mathrm{it}}+b_{0i}+b_{1i}\times {\text{time}}_{\mathrm{it}}+\varepsilon_{\mathrm{it}},$$

where $y_{\mathrm{it}}$ is the response of the ith subject at time point t, and ${\text{time}}_{\mathrm{it}}$ denotes time since baseline, $\beta_{0}$ is the intercept, $\beta_{1}$ is the time effect, $\beta_{2}$ is the interaction between treatment and time, $b_{0i}$ is the random intercept for the ith subject, $b_{1i}$ denotes the random slope of time for the ith subject, and $\varepsilon_{\mathrm{it}}$ is the error term. The model is a constrained longitudinal data analysis (cLDA) model with the baseline outcome measure included in the response vector and the baseline mean constrained to be the same across treatment groups,^12^ and additional covariates can be also included in the above model.

In the analysis of clinical trials, the LMM can also be fitted with time treated as categorical, and the treatment effect is then typically tested at the last visit. The borrowing methods considered in this study are also applicable in this setting.

## 3 THE POWER PRIOR: A BRIEF REVIEW

Ibrahim and Chen^6^ suggested to downweight the historical information by raising the historical likelihood to a power parameter, which generates the so‐called “power prior”. One possibility is to fix the power in advance, the posterior distribution of the parameters in the analysis model is then given by:

(3) $$p\left(\theta |y,y_{0},\alpha \right)\propto L\left(\theta |y\right)\;L{\left(\theta |y_{0}\right)}^{\alpha }\;p_{0}\left(\theta \right),$$

where θ is the set of parameters in the model, y denotes the current data, $y_{0}$ stands for the historical control data, and α is the power parameter. $L\left(\theta |y\right)$ is the current likelihood, $L\left(\theta |y_{0}\right)$ is the historical likelihood, and $p_{0}\left(\theta \right)$ is the prior for θ. The power parameter can be regarded as a weight parameter of the historical data and is restricted to the interval [0, 1], that is, 0≤α≤1. The value of α determines the amount of historical data to be borrowed in the analysis of the current data. When α=0, the power prior will not rely on the historical data, that is, no incorporation of the historical data, whereas the historical data has the same weight as the current data if α=1. The power parameter allows to control the influence of the historical data on the analysis of current data. In practice, one can evaluate the impact of choosing a particular fixed power by conducting a sensitivity analysis with different fixed power values.

Alternatively, one can treat the power parameter as a random variable and give it also a prior. Duan et al. proposed the MPP where the power prior distribution is normalized with a scaling constant.^9^ The general formulation of the posterior distribution in the MPP is:

(4) $$p\left(\theta ,\alpha |y,y_{0}\right)\propto L\left(\theta |y\right)\;\frac{L{\left(\theta |y_{0}\right)}^{\alpha }\;p_{0}\left(\theta \right)}{\int L{\left(\theta |y_{0}\right)}^{\alpha }\;p_{0}\left(\theta \right)d\theta }\;p_{0}\left(\alpha \right),$$

where the power prior is normalized with the scaling constant $\int L{\left(\theta |y_{0}\right)}^{\alpha }\;p_{0}\left(\theta \right)d\theta$ to satisfy the likelihood principle.^9^, ^13^

Until now, the MPP has mostly been implemented for univariate endpoints. For instance, Duan et al. implemented the MPP in water quality comparison between sites involving binary and Gaussian outcomes.^8^, ^9^ The method was later implemented in survival outcomes.^10^ In this article, we will extend the MPP to longitudinal data analysis based on linear mixed models.

## 4 THE IMPLEMENTATION OF THE MPP IN LONGITUDINAL DATA ANALYSIS

In this section, the motivation of the MPP implementation for a longitudinal data analysis and the technical details of its implementation in the LMM are discussed.

### 4.1 Motivation

There are two versions of the linear mixed model. The first is the hierarchical version, which specifies the distribution of the Gaussian response in two stages: the distribution of the response given the random effects and the distribution of the random effects. This leads to the conditional version of the LMM likelihood, so it is the likelihood of the observed data given the random effects, that is, $y_{i}|b_{i}∼N\left(X_{i}\beta +Z_{i}b_{i},R_{i}\right)$. This conditional version of the LMM is extensively used in Bayesian software, because the data augmentation algorithm (augmenting the data with the latent random effects) is straightforward. The second version is the LMM likelihood integrated over the random effects. For the LMM, the integration leads to an analytical expression, that is, $y_{i}∼N\left(X_{i}\beta ,Z_{i}{\mathrm{GZ}}_{i}^{T}+R_{i}\right)$, which is called the marginal likelihood. Because of the existence of an analytical expression of the marginal likelihood, this method is easy to implement in any kind of software, Bayesian or frequentist. In classical frequentist software one makes use of the marginal likelihood. These two versions of the LMM, the conditional (or hierarchical) and the marginal,^14^ lead to two different implementations of the MPP with either the conditional likelihood or the marginal likelihood raised to the power parameter.

Different implementations of the power prior in the generalized linear mixed model (GLMM) were first described by Ibrahim and Chen, albeit without the scaling constant to normalize the posterior.^6^ They pointed out that the power prior can be constructed by either exponentiating the historical likelihood given the random effects or the marginal historical likelihood after integrating out the random effects. In our study, the implementation of the MPP in the LMM is of particular interest.

For a fixed power parameter α, the power prior generated by raising the historical likelihood given the random effects to the power parameter is given by:

(5) $$p\left(\theta |y_{0},\alpha \right)\propto \int L{\left(\theta ,b_{0}|y_{0}\right)}^{\alpha }\;p\left(b_{0}|\theta \right)d\;b_{0}\;p_{0}\left(\theta \right),$$

where θ includes regression coefficients (β), the covariance matrix of the random effects (G), and the error variance ($\sigma^{2}$), $b_{0}$ denotes the historical random effects. The power prior constructed with the marginal historical likelihood is:

(6) $$p\left(\theta |y_{0},\alpha \right)\propto {\left[\int L\left(\theta ,b_{0}|y_{0}\right)\;p(b_{0}|\theta )d\;b_{0}\right]}^{\alpha }\;p_{0}\left(\theta \right).$$

It is also possible to define two versions of the MPP for LMMs depending on how we deal with the random effects, and we refer to these versions as the conditional MPP and the marginal MPP. The details of their implementation are discussed in the following subsections.

### 4.2 The conditional MPP

In the conditional MPP for a LMM, the power prior is obtained by raising the conditional historical likelihood to α, which is given by:

(7) $$p\left(\theta ,b_{0},\alpha |y_{0}\right)=\frac{L{\left(\theta ,b_{0}|y_{0}\right)}^{\alpha }\;p\left(b_{0}|\theta \right)\;p_{0}\left(\theta \right)}{\int \int L{\left(\theta ,b_{0}|y_{0}\right)}^{\alpha }\;p\left(b_{0}|\theta \right)\;p_{0}\left(\theta \right)d\;b_{0}d\theta }\;p_{0}\left(\alpha \right).$$

The calculation of the scaling constant requires integration with respect to both θ and $b_{0}$, and an algorithm that facilitates its calculation is described in Section 4.4.

Suppose that there are n subjects in the current data with $m_{i}$ repeated measures in the ith subject, and $n_{0}$ subjects in the historical control arm with $m_{0i^{\prime }}$ repeated measures in the $i^{\prime }$th subject. The joint posterior distribution of model parameters and the power parameter based on model (1) is:

$$\begin{array}{c} p\left(\beta_{C},\beta_{T},G,\sigma^{2},b,b_{0},\alpha |y,y_{0}\right)\propto \left[\prod_{i=1}^{n}p\left(y_{i}|\beta_{C},\beta_{T},b_{i},\sigma^{2}\right)\;p(b_{i}|G)\right]\;p_{0}\left(\beta_{T}\right)\times \\ \frac{\left[\prod_{i^{'}=1}^{n_{0}}p{\left(y_{0i^{'}}|\beta_{C},b_{0i^{'}},\sigma^{2}\right)}^{\alpha }\;p(b_{0i^{\prime }}|G)\right]\;p_{0}\left(\beta_{C}\right)\;p_{0}\left(G\right)\;p_{0}\left(\sigma^{2}\right)}{\int \int \left[\prod_{i^{'}=1}^{n_{0}}p{\left(y_{0i^{'}}|\beta_{C},b_{0i^{\prime }},\sigma^{2}\right)}^{\alpha }\;p(b_{0i^{\prime }}|G)\right]\;p_{0}\left(\beta_{C}\right)\;p_{0}\left(G\right)\;p_{0}\left(\sigma^{2}\right)d\;b_{0}d\theta }\;p_{0}\left(\alpha \right), \end{array}$$

where $\beta_{C}$ is a $\left(p-1\right)\times 1$ vector of fixed effects including common fixed effects in both the current data and the historical control arm, for instance, the intercept, the time effect, and $\beta_{T}$ is the treatment effect only in the current data, $b=\left(b_{1},\ldots ,b_{n}\right)$ denotes the current random effects, $b_{0}=\left(b_{01},\ldots ,b_{0n_{0}}\right)$ is the historical random effects, $y=\left(y_{1},\ldots ,y_{n}\right)$ stands for the current data, $y_{0}=\left(y_{01},\ldots ,y_{0n_{0}}\right)$ is the historical data, θ denotes model parameters including $\beta_{C}$, G, and $\sigma^{2}$.

The conditional MPP is an example of a partial discounting power prior in that the historical likelihood $p\left(y_{0i^{\prime }}|\beta_{C},b_{0i^{\prime }},\sigma^{2}\right)$ is downweighted whereas the distribution of the subject‐specific random effects $p\left(b_{0i^{\prime }}|G\right)$ is not.^3^ The difference between the partial discounting power prior in Ibrahim et al's paper^3^ and the conditional MPP in this study is that the historical random effects $b_{0}$ are not integrated out in this study due to the lack of closed‐form solution. Instead, the conditional MPP treats $b_{0}$ as model parameters.

### 4.3 The marginal MPP

In the marginal MPP, the power prior constructed with the discounted marginal historical likelihood is given by:

(8) $$p\left(\theta ,\alpha |y_{0}\right)=\frac{{\left[\int L\left(\theta ,b_{0}|y_{0}\right)\;p(b_{0}|\theta )d\;b_{0}\right]}^{\alpha }\;p_{0}\left(\theta \right)}{\int {\left[\int L\left(\theta ,b_{0}|y_{0}\right)\;p(b_{0}|\theta )d\;b_{0}\right]}^{\alpha }\;p_{0}\left(\theta \right)d\theta }\;p_{0}\left(\alpha \right).$$

The joint posterior distribution of model parameters and the power parameter in the marginal MPP is then given by:

$$\begin{array}{c} p\left(\beta_{C},\beta_{T},G,\sigma^{2},\alpha |y,y_{0}\right)\propto \prod_{i=1}^{n}p\left(y_{i}|\beta_{C},\beta_{T},G,\sigma^{2}\right)\;p_{0}\left(\beta_{T}\right)\times \\ \frac{{\left[\prod_{i^{'}=1}^{n_{0}}p\left(y_{0i^{\prime }}|\beta_{C},G,\sigma^{2}\right)\right]}^{\alpha }p_{0}\left(\beta_{C}\right)\;p_{0}\left(G\right)\;p_{0}\left(\sigma^{2}\right)}{\int {\left[\prod_{i^{\prime }=1}^{n_{0}}p\left(y_{0i^{\prime }}|\beta_{C},G,\sigma^{2}\right)\right]}^{\alpha }p_{0}\left(\beta_{C}\right)\;p_{0}\left(G\right)\;p_{0}\left(\sigma^{2}\right)d\theta }\;p_{0}\left(\alpha \right), \end{array}$$

where $p\left(y_{0i^{\prime }}|\beta_{C},G,\sigma^{2}\right)$ is the marginal distribution of $y_{0i^{\prime }}$ averaged over the historical random effects $b_{0i^{\prime }}$. The implementation of the marginal MPP in linear mixed models can be straightforward, while its implementation in generalized linear mixed models can be computationally intractable because the integration with respect to the random effects is required in every iteration.

Unlike the conditional MPP, the marginal MPP is not a *partial discounting power prior* because the subject‐specific random effects are integrated out, and the whole marginal likelihood is discounted using the power parameter.

In the above implementation of the conditional MPP and the marginal MPP, the estimation of $\beta_{T}$ is not directly informed by its enhanced prior because only the historical control arm is considered. However, its estimation could still be improved with the power prior of other model parameters.

Moreover, a theoretical comparison between the conditional MPP and the marginal MPP for LMMs was conducted, but no closed‐form expressions for the marginal posterior of the power parameter are available to our knowledge. In a simplified comparison assuming known covariance matrix for random effects, G, and error variance, $\sigma^{2}$, the conditional MPP tends to borrow more than the marginal MPP given the same power value, which implies that two equal power values in both approaches have different downweighting for the historical information in this case. Please refer to Section 1 in the supplementary document for more details.

### 4.4 Estimation

According to the above formulations, posterior sampling in both the conditional MPP and the marginal MPP can be done only if the scaling constant is calculated. In data with univariate Gaussian or binary response, the scaling constant has a closed‐form expression.^7^, ^11^, ^15^ However, there is no closed‐form for the scaling constant in Cox models and LMMs.

For a proportional hazards model, van Rosmalen et al. have adopted a path sampling algorithm to calculate the scaling constant.^10^, ^16^ We have adapted this path sampling algorithm to the LMM. In short, the implementation of the MPP for a LMM can be divided into two steps as follows.

- Step 1: calculate the scaling constant using the path sampling algorithm. In this step, scaling constants corresponding to a grid of fixed power values are calculated via a path sampling algorithm.
- Step 2: sample from the posterior based on scaling constants calculated in Step 1 Since the power parameter is continuous in [0, 1], the scaling constants for powers not belonging to the grid are obtained via linear interpolation.

Sampling was conducted with Hamiltonian Monte Carlo (HMC) in Stan,^17^ details of the estimation procedure and the Stan syntax are given in the supplementary material S1.

Furthermore, the efficiency of the sampler in Step 2 of the conditional MPP is relatively low due to a large number of historical random effects to be sampled, which brings difficulty to the convergence of model parameters. To improve the efficiency of the sampler, we proposed a new sampler that can avoid sampling the historical random effects because they are not parameters of primary interest after all. Considering only linear mixed models are involved in the study, the historical random effects ($b_{0}$) can still be integrated out from the power prior after the historical likelihood, $L\left(\theta ,b_{0}|y_{0}\right)$, raised to the power parameter. Based on results of preliminary results, the new sampler is more efficient than the sampler with historical random effects in terms of (a) computational time and (b) number of iterations required to achieve convergence. The details of the new sampler in Step 2 of the conditional MPP can be found in Section 3 of the supplementary document.

According to the results of preliminary simulations, we have found that there may exist a bimodal posterior of the power parameter with certain level of between‐study heterogeneity in the conditional MPP. To achieve geometric ergodicity of the bimodal posterior, we set different random initial power values for the MCMC chains and a high target average proposal acceptance probability in Stan's adaptation period (from the default 0.8–0.95). The geometric ergodicity can be achieved with the above initialization strategy according to diagnostic statistics including Bayesian fraction of missing information, number of divergent transitions, and the Gelman‐Rubin convergence diagnostic $\hat{R}$_._ ^17^, ^18^, ^19^ The bimodality can make the convergence of the sampler difficult, thus researchers should be wary of distinguishing a convergence problem from a genuine bimodal posterior of the power parameter when implementing the conditional MPP.

## 5 ALTERNATIVE BORROWING METHODS IN LONGITUDINAL DATA ANALYSIS

Although relatively few alternative methods are available for historical borrowing in longitudinal settings, here we consider an application of the commensurate prior, as well as ignoring or pooling the historical data. Details of these methods are presented below.

Hobbs et al. proposed the commensurate prior that allows for the commensurability (comparability) between the historical and current data to determine the amount of historical information to be borrowed in the LMM.^2^, ^4^

Unlike the MPP, the commensurate prior allows for different parameters for the historical and current data. The assumption of the commensurate prior is that the parameters of interest in the current study (θ) come from a multivariate normal distribution centered on the corresponding parameters in the historical control arm ($\theta_{0}$), that is, $\theta ∼\mathrm{MVN}\left(\theta_{0},\sum \right)$ where ∑ is the covariance matrix of θ given $\theta_{0}$. The multivariate normal distribution can determine the amount of historical data to be incorporated in the current analysis.^4^

The LMMs for the current study and the historical control arm can be formulated as:

(9) $$y_{i}=X_{i}\;\beta_{C}+d_{i}\;\beta_{T}+Z_{i}\;b_{i}+\varepsilon_{i},$$

And,

(10) $$y_{0i^{\prime }}=X_{0i^{\prime }}\;\beta_{0C}+Z_{0i^{'}}\;b_{0i^{\prime }}+\varepsilon_{0i^{\prime }},$$

where $X_{i}$ is the design matrix of fixed effects for the ith subject in the current study and $X_{0i^{\prime }}$ is the design matrix of fixed effects for the $i^{'}$th subject in the historical control arm, and $\beta_{C}$ and $\beta_{0C}$ are the vector of fixed effects for the current study and the historical control arm respectively. $d_{i}$ is the treatment assignment of the ith subject in the current study ($d_{i}=1$ for treatment group, and $d_{i}=0$ for the control group). In Hobbs' paper, $d_{i}$ is the main treatment effect, whereas $d_{i}$ is the interaction between treatment and time in this study to be consistent with the model used in the MPP. $Z_{i}$ is the design matrix of random effects and $b_{i}$ denotes the vector of random effects for the ith subject in the current study, $b_{i}∼N\left(0,G\right)$ where G is the covariance matrix of the current random effects. $Z_{0i^{\prime }}$ denotes the design matrix of random effects and $b_{0i^{\prime }}$ denotes the vector of random effects for the $i^{'}$th subject, $b_{0i^{\prime }}∼N\left(0,G_{0}\right)$ where $G_{0}$ is the covariance matrix of the historical random effects. $\varepsilon_{i}∼N\left(0,\sigma^{2}I_{m_{i}}\right)$ is the random error for the ith subject in the current study, and $\varepsilon_{0i^{\prime }}∼N\left(0,\sigma_{0}^{2}I_{m_{0i^{\prime }}}\right)$ is the random error for the $i^{'}$th subject in the historical study.

Based on the above two LMMs, the posterior distribution of model parameters for the commensurate prior is given by:

$$\begin{array}{c} p\left(\beta_{C},\beta_{0C},\beta_{T},G,G_{0},\sigma^{2},\sigma_{0}^{2},\sum_{\beta_{C}}|y,y_{0}\right)\propto \\ \left[\prod_{i=1}^{n}p\left(y_{i}|\beta_{C},\beta_{T},G,\sigma^{2}\right)\right]\;p\left(\beta_{C}|\beta_{0C},\sum_{\beta_{C}}\right)\;\left[\prod_{i^{'}=1}^{n_{0}}p\left(y_{0i^{\prime }}|\beta_{0C},G_{0},\sigma_{0}^{2}\right)\right]\times \\ p_{0}\left(\beta_{0C}\right)\;p_{0}\left(\beta_{T}\right)\;p_{0}\left(G\right)\;p_{0}\left(G_{0}\right)\;p_{0}\left(\sigma^{2}\right)\;p_{0}\left(\sigma_{0}^{2}\right)\;p_{0}\left(\sum_{\beta_{C}}\right), \end{array}$$

where $p\left(\beta_{C}|\beta {'}_{0C},\sum_{\beta_{C}}\right)$ is the multivariate normal commensurate prior for $\beta_{C}$, $\sum_{\beta_{C}}$ is a $\left(p-1\right)\times \left(p-1\right)$ covariance matrix of $\beta_{C}$ given $\beta_{0C}$. Note that the marginal likelihood of the LMM is used in the above model as Hobbs et al. did. The conditional likelihood could also be used in the commensurate prior, but this option was not explored in this study.

There are also two other choices to deal with the historical control arm when analyzing the current data, which are no borrowing and pooling. In our study, the no borrowing and pooling method use the same Bayesian LMM, and the posterior distributions of the model parameters is given by:

(11) $$p\left(\beta_{C},\beta_{T},G,\sigma^{2}|y\right)\propto \prod_{i=1}^{n}p\left(y_{i}|\beta_{C},\beta_{T},G,\sigma^{2}\right)\;p_{0}\left(\beta_{C}\right)\;p_{0}\left(\beta_{T}\right)\;p_{0}\left(G\right)\;p_{0}\left(\sigma^{2}\right),$$

where the priors of the parameters, that is, $p_{0}\left(\beta_{C}\right)$, $p_{0}\left(\beta_{T}\right)$, $p_{0}\left(G\right)$, $p_{0}\left(\sigma^{2}\right)$, are typically chosen to be noninformative.^20^

## 6 SIMULATION STUDY

The simulation study was conducted to assess the performance of the conditional MPP and the marginal MPP in a longitudinal data analysis and to compare with the abovementioned alternative methods. In the remainder of the section, the generation of the simulated data, the settings and details of the simulation study, for example, the analysis model, the priors for the parameters, execution of the simulation, and simulation results are presented.

### 6.1 The generation of the simulated data

The simulation study was based on a two‐arm RCT with longitudinal outcomes, where one historical control arm was available. The analysis model in the simulation study is given by,

(12) $$y_{\mathrm{itj}}=\beta_{0}+\beta_{1}\times {\text{time}}_{\mathrm{itj}}+\beta_{2}\times {\mathrm{trt}}_{\mathrm{ij}}\times {\text{time}}_{\mathrm{itj}}+b_{0\mathrm{ij}}+b_{1\mathrm{ij}}\times {\text{time}}_{\mathrm{itj}}+\varepsilon_{\mathrm{itj}},$$

where $y_{\mathrm{itj}}$ is the outcome of the ith subject at time point t for the jth study (j=1 for the current study, j=2 for the historical control arm), $\beta_{0}$ is the intercept, $\beta_{1}$ is the time effect, $\beta_{2}$ is the interaction between treatment and time, ${\mathrm{trt}}_{\mathrm{ij}}$ = 0 or 1 when j=1 depending on the treatment allocation, and ${\mathrm{trt}}_{\mathrm{ij}}=0$ when j=2. $b_{0\mathrm{ij}}$ is the random intercept for the ith subject at the jth study, $b_{1\mathrm{ij}}$ is the random slope for time trend for the ith subject in the jth study, and $b_{\mathrm{ij}}∼N\left(0,G\right)$, $\varepsilon_{\mathrm{itj}}$ is the error term, and $\varepsilon_{\mathrm{itj}}∼N\left(0,\sigma^{2}\right)$. For the MPP, the subjects have a common intercept ($\beta_{0}$) and time effect ($\beta_{1}$), and subjects in the current treatment arm also have a treatment effect ($\beta_{2}$). The subject‐specific random effects parameterize the between‐subject heterogeneity. In the commensurate prior, the parameters including the regression coefficients, the covariance matrix and the error variance are allowed to differ between studies. Note that the treatment effect is not included in the historical likelihood in that only information from the historical control arm is considered.

In the simulation study, we sampled the historical and the current control parameters from a common multivariate normal distribution. The reason for sampling control parameters was that we want to evaluate the performance of different approaches averaged over a range of historical parameters instead of a particular fixed historical trial. This type of data generating process was also used in previous studies that assess different historical borrowing methods.^10^, ^11^, ^21^ Therefore, in the simulation of the data, we have included study‐specific random effects for the intercept ($\beta_{0}$) and the time effect ($\beta_{1}$) to represent the between‐study heterogeneity. The simulated data sets were generated according to,

(13) $$y_{\mathrm{itj}}=\beta_{0}+\beta_{1}\times {\text{time}}_{\mathrm{itj}}+\beta_{2}\times {\mathrm{trt}}_{\mathrm{ij}}\times {\text{time}}_{\mathrm{itj}}+b_{0\mathrm{ij}}+b_{1\mathrm{ij}}\times {\text{time}}_{\mathrm{itj}}+d_{0j}+d_{1j}\times {\text{time}}_{\mathrm{itj}}+\varepsilon_{\mathrm{itj}},$$

where $d_{0j}$ is the study‐specific intercept and $d_{1j}$ is the study‐specific time effect to model the between‐study heterogeneity, and $d_{j}∼N\left(0,T\right)$ where T is a 2×2 covariance matrix for the study‐specific random effects, which models the between‐study heterogeneity.

In the simulation study, T was chosen to be a diagonal matrix with diagonal elements as $\sigma_{d_{0}}^{2}$ and $\sigma_{d_{1}}^{2}$. Seven scenarios with four levels of between‐study heterogeneity were considered, we considered them to be no heterogeneity ($\sigma_{d_{0}}^{2}=\sigma_{d_{1}}^{2}=0$), low/moderate/high heterogeneity with only a between‐study random intercept ($\sigma_{d_{0}}^{2}\neq 0$, $\sigma_{d_{1}}^{2}=0$), and low/moderate/high heterogeneity with between‐study random intercept and slope ($\sigma_{d_{0}}^{2}\neq 0$, $\sigma_{d_{1}}^{2}\neq 0$). Different T matrices corresponding to different levels of the between‐study heterogeneity are given in Table 1. The levels of between‐study heterogeneity were abbreviated as “No”, “RI + Low”, “RI + Moderate”, “RI + High”, “RIS + Low”, “RIS + Moderate”, and “RIS + High” respectively where “RI” represents that there is only between‐study random intercept and “RIS” represents that there are between‐study random intercept and slope.

**TABLE 1:** The covariance matrix of the between‐study random effects for different between‐study heterogeneity levels

Random effects	Heterogeneity level	$\sigma_{d_{0}}^{2}$	$\sigma_{d_{1}}^{2}$	Scenario
No	No	0	0	No
Intercept	Low	0.01	0	RI + Low
Intercept	Moderate	0.09	0	RI + Moderate
Intercept	High	0.16	0	RI + High
Intercept + Slope	Low	0.01	0.01	RIS + Low
Intercept + Slope	Moderate	0.09	0.09	RIS + Moderate
Intercept + Slope	High	0.16	0.16	RIS + High

The number of subjects per arm for historical and current data was 100, and the number of repeated measurements was six. We let the time variable vary from 0 to 1 with increments of 0.2. The parameters in the data generation model are: $\beta_{0}=2$, $\beta_{1}=1$, $\sigma_{b_{0}}^{2}=0.25$, $\sigma_{b_{1}}^{2}=0.25$, $\sigma_{b_{0},b_{1}}=0$, $\sigma_{\varepsilon }^{2}=1$. The interaction is zero ($\beta_{2}=0$) for scenarios without treatment effect, and $\beta_{2}=0.36$ for scenarios with treatment effect. A full factorial simulation study that combines seven different levels of between‐study heterogeneity with two choices of treatment effect (i.e., 14 scenarios in total) was conducted.

Five methods including (a) no borrowing of the historical data, (b) the conditional MPP, (c) the marginal MPP, (d) the commensurate prior based on the marginal LMM, and (e) the pooling analysis were compared and evaluated with 500 replications. All simulations were conducted with the software Stan version 2.19.1. When simulating the methods: no borrowing, pooling and the commensurate prior, four chains were initiated, and 2000 iterations were run with 1000 burn‐in iterations per chain. For the conditional MPP and the marginal MPP, the fixed power values in Step 1 ranged from 0 to 1 with an increment of 0.02. Each power value had one chain with 100 iterations, and the posterior sampling in Step 2 was based on four chains, each chain had 2000 iterations with 1000 burn‐in iterations.

The priors of the parameters in the simulation were taken as follows. The prior for the regression coefficients is the g‐prior, that is, $N\left(0,g\sigma^{2}{\left(X^{T}X\right)}^{-1}\right)$ with g equals the number of observations in the current study.^20^ The covariance matrix of the random effects (G) is decomposed into a vector of *SD*s of the random effects (τ) and a correlation matrix (Ω), that is, $G=\mathrm{diag}\left(\tau \right)\times \Omega \times \mathrm{diag}\left(\tau \right)$. The prior for the components in τ is half‐normal (0, 1) and the prior for the correlation matrix is $\mathrm{LKJ}\left(\eta \right)$ with η=1_._ ^22^ Note that the prior for G in this simulation study differs from the inverse Wishart prior used by Hobbs et al. in the commensurate prior, but the modification is unlikely to change the estimation of the treatment effect. The prior for the *SD* of the error term is half‐normal (0, 4). Additionally, the covariance matrix of $\beta_{C}$ conditional on $\beta_{0C}$ in the commensurate prior ($\sum_{\beta_{C}}$) was also decomposed into a vector of *SD*s ($\sigma_{\beta_{0}}$, $\sigma_{\beta_{1}}$) and a correlation matrix. The prior for *SD*s is half‐normal (0, 1) and the prior for the correlation matrix is again $\mathrm{LKJ}\left(1\right)$. The prior for the power parameter is Beta (1) for both the conditional MPP and the marginal MPP.

The parameter of interest is the treatment effect, that is, the interaction between treatment and time. The methods were evaluated in terms of hypothesis testing and effect estimation. The treatment effect $\beta_{2}$ is statistically significant if the 95% credible interval does not contain the value 0. The type I error rate and statistical power were defined based on the above decision rule. Bias, posterior *SD*, and mean squared error (MSE) of the estimated treatment effect for different methods were computed to measure effect estimation.

### 6.2 Results of the simulation study

All methods in the simulation achieved good convergence under the above settings with $\hat{R}$ in all simulated data sets less than 1.05. The conditional MPP is the most time‐consuming among the methods, which took approximately 9 min for each simulated data set with the above setting. The computational time of the marginal MPP was about 3 min for each simulation.

**FIGURE 1:**
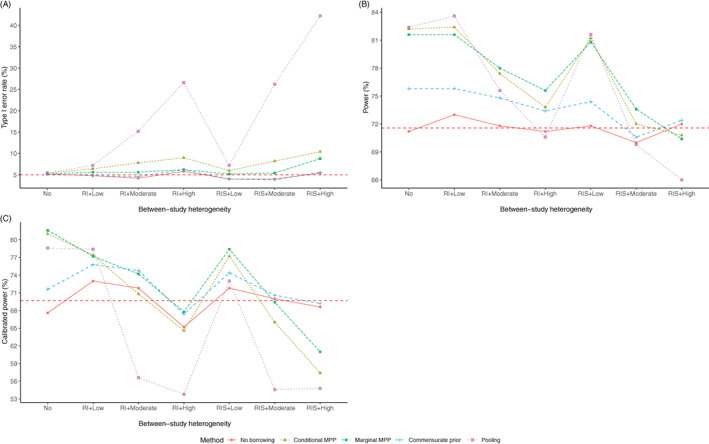
The type I error rate (A), statistical power (B), and calibrated power (C) of the estimated treatment effect for different methods based on 500 simulated data sets

The type I error rates and statistical powers of the methods considered in the simulation are visualized in Figure 1, where the dashed line in Figure 1A is the nominal 5% type I error rate and the dash line in Figure 1B is the average of statistical powers of “No borrowing” method in different scenarios.

Pooling the current data and historical control arm yielded a power gain of 11.2% compared to “No borrowing” when there was no between‐study heterogeneity. However, the method had a type I error rate ranged from 7.2% to 42.2% in different scenarios with between‐study heterogeneity, which was higher than the nominal type I error rate of 5%. The conditional MPP borrowed the most historical information among the dynamic borrowing methods. This method led to a type I error rate from 7.8% to 10.4% with moderate or high between‐study heterogeneity, although it had a power gain (9.4%–11.0%) with no or low between‐study heterogeneity. The marginal MPP had a type I error rate of 8.8% in the “RIS + High” scenario, but the method yielded a power gain (3.6%–10.4%) in scenarios other than “RIS + High” with the type I error rate ranging from 5.2% to 6.2%. A McNemar test was conducted to test the difference of type I error rates in the conditional and the marginal MPP in each scenario because the same 500 simulated data sets were analyzed using the two approaches, and statistically significant difference was found in “RI + Moderate”, “RI + High”, “RIS + Moderate”, and “RIS + High” scenarios. The commensurate prior had the type I error rate close to the nominal 5% rate with different levels of between‐study heterogeneity (3.8%–6.2%). Although the approach yielded less power gain than the marginal MPP expect in “RIS + High” scenario (0.6%–4.6% vs. 3.6%–10.4%), it was the only borrowing method that had higher statistical power compared to “No borrowing” in all scenarios. The decreased statistical power for the conditional MPP and marginal MPP in “RIS + High” scenario shows that borrowing an excessive amount of information from highly heterogeneous historical controls may harm the statistical power. The exact values of type I error rate and statistical power and the corresponding Monte Carlo *SE*s for all methods are given in Table S1.

To improve the comparison of the statistical power, we calculated the “calibrated” power by fixing the type I error rate of all the methods to 5%, that is, adjusting the coverage probability of the credible interval for hypothesis testing in scenarios without treatment effect. The calibrated power would allow fair comparison across different methods in terms of statistical power. Calibrated powers are presented in Figure 1C, where the dashed line is the average calibrated power of “No borrowing” method in different scenarios. As can be seen from the figure, the “Pooling” method performed poorly when the between‐study heterogeneity was moderate to high. The conditional MPP also performed worse than “No borrowing” when the between‐study heterogeneity was moderate to high, the difference was especially large when the between‐study heterogeneity was “RIS + Moderate” and “RIS + High”. The marginal MPP yielded more or comparable power compared to “No borrowing” except in “RIS + High” scenario, while the commensurate prior performed better than “No borrowing” in terms of power in all scenarios although its power gain was limited when the between‐study heterogeneity was low. The above conclusions drawn from Figure 1C validated those drawn from Figure 1A,B.

Bias, posterior *SD* and MSE of the estimated regression coefficients along with the corresponding Monte Carlo *SE* in the simulation study are presented in Tables S2–S4. Table S2 shows that all methods considered resulted in an unbiased estimated regression coefficient for treatment. Table S3 presents posterior *SD*s of the estimated treatment effect in the methods. The posterior *SD* depicts the precision of the estimated treatment effect, and we use this parameter as a measure of the amount of historical borrowing. As can be seen from the table, the conditional MPP borrowed most of the historical information, while the commensurate prior borrowed the least of the historical information in all scenarios. The average MSEs in Table S4 represent how much the estimation of the treatment effect can be improved by incorporating the historical information in these borrowing methods. In scenarios with no or low between‐study heterogeneity, “No borrowing” had the highest MSE and thus borrowing historical data can improve the estimation. Pooling the current data and the historical controls produced the greatest MSE with moderate and high between‐study heterogeneity. The MSE of the conditional MPP was similar to that for “No borrowing” in “RI + moderate” scenario, and the MSE for the approach was worse in “RI + High”, “RIS + Moderate” and “RIS + High” scenarios. The marginal MPP and the commensurate prior had similar MSEs as for “No borrowing” when the between‐study heterogeneity was moderate or high, which means that not much gain can be expected in improving the estimation of the treatment effect. Monte Carlo SEs of the above performance measures are all acceptable, which indicates low simulation uncertainty and thus valid simulation results.

**FIGURE 2:**
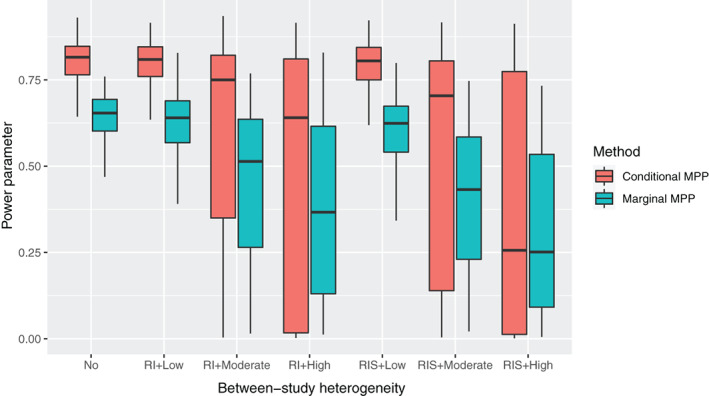
Box plots for the posterior means of the power parameter in the conditional MPP and the marginal MPP with different levels of between‐study heterogeneity based on 500 simulated data sets

Distributions of 500 posterior means of the power parameter for the conditional MPP and the marginal MPP in simulation scenarios without treatment effect are visualized with box plots in Figure 2, where the dots are means of the posterior means in different scenarios. Both MPP approaches can incorporate the historical information adaptively accounting for the difference between the current and historical data. Note that power parameters in the conditional MPP and the marginal MPP have different interpretations (see Section 1 of the supplementary document), the amount of borrowing can be quantified with the variability of the treatment effect in a more straightforward way. Medians and interquartile ranges (IQR) of the posterior means of the power parameter for these two approaches in all simulation scenarios are presented in Table S5. In addition, posterior modes of the power parameter in both the conditional MPP and the marginal MPP were 1 with no between‐study heterogeneity, which was in line with the finding in Duan et al.'s study.^9^

Due to inflated type I error rates yielded in the conditional MPP and the marginal MPP, we also conducted a sensitivity analysis for the MPP with a more skeptical prior, Beta (1, 2), for the power parameter. The estimates for the power parameter were lower than those obtained from a Beta (1) prior, and the type I error rates were slightly reduced in some scenarios. However, the Beta (1, 2) prior did not control the type I error rate either in scenarios that had inflated type I error rates with the uniform prior. Details of the sensitivity analysis can be found in Tables S6 and S7.

## 7 THE MPP IN PRACTICE: THE ALZHEIMER'S DISEASE COOPERATIVE STUDY

In addition to the simulation study, it is also worthwhile to implement the dynamic borrowing methods in the analysis of real clinical trial data to further evaluate their performance in practice.

The motivating data sets were obtained from the University of California, San Diego Alzheimer's Disease Cooperative Study (ADCS) Legacy database. The ADCS includes a series of clinical studies addressing treatments for both cognitive and behavioral symptoms in Alzheimer's disease.

Among these clinical studies, two studies were chosen to be the historical and current data set respectively based on their similarity, namely ADC‐016 and ADC‐027.^23^, ^24^ Both studies are multicenter, randomized, double‐blind, placebo‐controlled trials, conducted in the United States, and the inclusion criteria for both of the studies were: age older than 50 years, and a Mini‐Mental State Examination (MMSE) score^25^ within the range of 14 to 26. In ADC‐016, the researchers evaluated the effects of high dose B vitamins on cognitive decline of Alzheimer's disease, the primary outcome was the Alzheimer's Disease Assessment Scale‐Cognitive (ADAS‐cog) score,^26^ which was measured from baseline to month 18 every 3 months (7 repeated measurements). In the ADC‐027 study, researchers investigated the effect of Docosahexaenoic Acid (DHA) supplement on Alzheimer's disease with ADAS‐cog score measured four times during the study period. The patients were examined at baseline and then every 6 months until month 18. The baseline characteristics of these two studies are given in Table 2.

**TABLE 2:** The baseline characteristics of the candidate studies

Study	ADC‐016	ADC‐027
Number of subjects	409 (T: 240, P: 169)	402 (T: 238, P: 164)
Study period	2003–2006	2007–2009
Study duration (months)	18	18
Baseline age (mean [*SD*])	76.3 (8.0)	76 (8.7)
Sex (% of female)	56.0	52.2
Years of education (mean [*SD*])	13.9 (3.1)	14 (2.8)
Baseline MMSE (mean [*SD*])	21.0 (3.5)	20.7 (3.6)

As can be seen from the baseline characteristics, the allocation ratio of ADC‐027 is 3:2 with fewer patients randomized to the control arm, which makes it more appealing to borrow the historical control arm in the current analysis to supplement the number of subjects in the control arm.

The data were analyzed with a Bayesian LMM with ADAS‐score as outcome, and the analysis was an intent‐to‐treat analysis including all randomized patients regardless of the missingness of the outcome as in the original article. The model was also a cLDA model, and covariates included the model were baseline age, sex, years of education, baseline MMSE score, time, and the interaction between treatment and time (i.e., treatment effect). The main effect of treatment was excluded due to randomization. In addition, a subject‐specific random intercept and a random linear time effect were included as random effects. The parameters of interest were the time effect and the interaction, and the statistical significance of these effects was also based on the 95% credible intervals. The chosen model is,

$$\begin{array}{c} {\text{ADAS}}_{\mathrm{itj}}=\;\beta_{0}\;+\;\beta_{1}\;\times \;{\mathrm{age}}_{\mathrm{ij}}\;+\;\beta_{2}\;\times \;{\mathrm{sex}}_{\mathrm{ij}}\;+\;\beta_{3}\;\times \;{\text{education}}_{\mathrm{ij}}\;+\;\beta_{4}\;\times {\text{MMSE}}_{\mathrm{ij}}\;+\;\\ \beta_{5}\times {\text{time}}_{\mathrm{itj}}+\beta_{6}\times {\text{treatment}}_{\mathrm{ij}}\times {\text{time}}_{\mathrm{itj}}+\;b_{0\mathrm{ij}}+\;b_{1\mathrm{ij}}\times \;\text{time}\;+\;\varepsilon_{\mathrm{itj}}, \end{array}$$

where ${\text{ADAS}}_{\mathrm{itj}}$ is the ADAS‐cog score for the ith subject at time point t in the jth study (j=1 for the historical control arm and j=2 for the current study).

The priors of the parameters, estimation method, and statistical software were the same as the settings in the simulation study, but each chain of the four chains had 5000 iterations with 2500 burn‐in iterations (10,000 post‐warmup samples in total) to achieve convergence and get more precise estimates in this more complicated model. Parameter estimates (posterior means) of different borrowing methods and their 95% credible intervals (CI) are presented in Table 3.

**TABLE 3:** Parameter estimates of the ADC‐027 trial using different borrowing methods

Method	Time effect $\beta_{5}$			Treatment effect $\beta_{6}$		
	Posterior mean	Posterior *SD*	95% CI	Posterior mean	Posterior *SD*	95% CI
No borrowing	0.521	0.039	(0.444, 0.596)	−0.022	0.051	(−0.121, 0.079)
Conditional MPP	0.462	0.026	(0.411, 0.514)	0.034	0.040	(−0.045, 0.112)
Marginal MPP	0.477	0.032	(0.416, 0.542)	0.022	0.046	(−0.068, 0.109)
Commensurate prior	0.516	0.039	(0.441, 0.592)	−0.015	0.050	(−0.117, 0.081)
Pooling	0.459	0.025	(0.411, 0.508)	0.039	0.040	(−0.039, 0.116)

**FIGURE 3:**
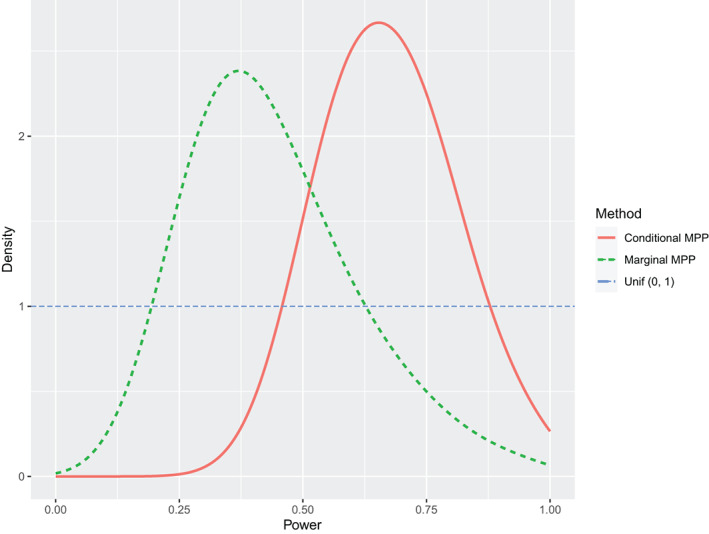
Posterior distributions of the power parameters in the conditional MPP and the marginal MPP in the analysis of ADCS data

The estimate of the time effect in the historical control arm is 0.410 (Posterior *SD* = 0.029, 95% CI: 0.352–0.468). The time effect estimate based on the data of the ADC‐027 trial only is 0.521 (Posterior *SD* = 0.039, 95% CI: 0.444–0.596), while the time effect estimate is 0.459 (Posterior *SD* = 0.025, 95% CI: 0.411–0.508) by pooling the ADC‐027 and the control arm of the ADC‐016. The time effect estimates in the dynamic borrowing methods lie between 0.459 and 0.521. The interaction between treatment and time is −0.022 (Posterior *SD* = 0.051, 95% CI: −0.121 to 0.079) when based on the data of the ADC‐027 only and its estimate is 0.039 (Posterior *SD* = 0.040, 95% CI: −0.039 to 0.116) when pooling the data of the ADC‐027 and the ADC‐016. The estimates for the time effect and the interaction for the ADC‐027 trial are slightly different from those in the original article due to different covariates included in the model. The estimates of the interaction term from the dynamic borrowing methods also range between the estimates from the “No borrowing” and “Pooling” method. The estimates of the conditional MPP are closest to those from the pooling method, while the estimates of the commensurate prior are furthest to those from the pooling method, which indicates that the conditional MPP borrows the most of the historical information and the commensurate prior incorporates the least of it. The medians and IQRs of the power parameters in the conditional MPP and the marginal MPP are 0.67 (0.58, 0.76) and 0.41 (0.32, 0.53) respectively, and their distributions are visualized in Figure 3.

**FIGURE 4:**
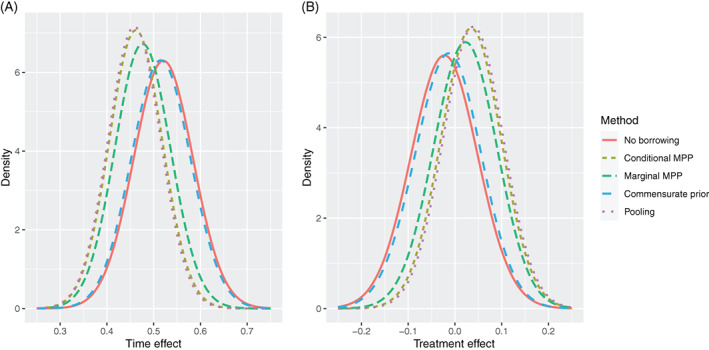
Posterior distributions of time effect (A) and treatment effect (B) of different methods in the analysis of ADCS data

The posterior distributions of the time effect and the treatment effect in model (17) are presented in Figure 4. The posterior distributions of the parameters are in accordance with the results presented above. The conditional MPP borrows more historical information than other methods do, and the commensurate prior tends to incorporate the smallest amount of the historical information.

Including the historical information can change the direction of the treatment effect, as is shown in the results of the conditional MPP, the marginal MPP and the “Pooling” method. However, the treatment effect ($\beta_{6}$) estimate is not statistically significant for any method. Our analyses confirm that DHA, the investigational treatment arm in ADC‐027, has no significant effect on the cognitive function of Alzheimer's disease patients, as also obtained with the original data.^24^ Although the substantive conclusions would be unchanged, the dynamic borrowing methods improve the precision of the estimates.

## 8 DISCUSSION

Dynamic borrowing methods have seldom been implemented in the analysis of clinical trials with longitudinal outcomes. It can be desirable to take advantage of the merits of dynamic borrowing methods, for example, increased statistical power, in longitudinal data analysis, and our study first implemented the MPP in this context. The results of the study have shown that the marginal MPP and the commensurate prior are viable methods to incorporate historical controls in the analysis of clinical trials with longitudinal outcomes, while the conditional MPP tends to borrow excessive historical information even if the historical and current study are rather incomparable. Pooling the current data and historical controls can inflate the type I error rate when there was between‐study heterogeneity. The observed data typically provide only limited information on the amount of between‐study heterogeneity, and the type I error rate tends to increase with the amount of heterogeneity. This means we should only adopt dynamic borrowing methods where a large level of heterogeneity can be ruled out a priori, based on comparability criteria, or alternatively we should use borrowing methods that give good results across a wide range of heterogeneity levels.

The conditional MPP increases the statistical power with at most a small increase of the type I error rates in scenarios with no or low between‐study heterogeneity, however it yields a type I error rate higher than 7% when the between‐study heterogeneity is moderate or high, which indicates that the conditional MPP cannot prevent the prior‐data conflict efficiently. Both the marginal MPP and commensurate prior can yield type I error rates close to 5% if the between‐study heterogeneity is not excessively high, but the marginal MPP has more power gain than the commensurate prior does in these scenarios. The commensurate prior yields the largest posterior *SD*s of estimated treatment effect, which indicates that the commensurate prior is more conservative than both the conditional MPP and the marginal MPP. The marginal MPP is a viable method to incorporate historical data if the between‐study heterogeneity is not extra high, for instance, “RIS + High” in this study. The commensurate prior is a robust method for relatively heterogeneous historical controls, although its power gain is limited even with relatively low between‐study heterogeneity. Note that the conclusion on the performance of the commensurate prior is drawn based on the specific prior for the between‐study covariance matrix ($\sum_{\beta_{C}}$), a more optimistic prior for $\sum_{\beta_{C}}$ may lead to more power gain. Nonetheless the prior used for $\sum_{\beta_{C}}$ in this study was sensible and realistic because it was specified based on the data. Besides, the commensurate prior did not incorporate information of (co)variance parameters, which may lead to less precise treatment effect estimate. Schmidli et al. has implemented the meta‐analytic‐predictive approach to incorporate historical information of variance parameters,^27^ it may also be possible to extend the commensurate prior to incorporate historical information of (co)variance parameters.

The major advantage of incorporating historical data in the analysis of the current data is the power gain compared to “No borrowing”. However, the power gain might be accompanied by potential increases of the type I error rate. In scenarios with no or low between‐study heterogeneity, all dynamic borrowing methods can lead to the type I error rates close to 5%. In scenarios with moderate or high between‐study heterogeneity, the conditional MPP leads to an inflated type I error rate, whereas the marginal MPP has type I error rate close to 5% and higher statistical power with that of “No borrowing”. Therefore it is desirable to assess the between‐study heterogeneity before implementing the MPP to preclude a highly heterogeneous historical control arm using specific assessment tools.^28^ Moreover, a more skeptical prior than the Beta (1, 2) prior used in the sensitivity analysis for the power parameter may be considered to control the type I error rate, which implies that the MPP especially the conditional MPP is excessively liberal in incorporating the historical information. Although inflation of the type I error rate is common in the context of historical borrowing methods, regulatory authorities such as FDA do not totally forbid the use of historical borrowing methods due to strict control of the type I error rate. Instead, it is stated in an official guidance issued by FDA that “When using prior information, it may be appropriate to control type I error at a less stringent level than when no prior information is used.”.^29^ When there is a slightly inflated type I error rate using historical borrowing methods, researchers could still accept the inflation because the power gain may lead to benefits that outweigh the inflated type I error rate in a specific research situation.^30^, ^31^

In the simulation of the study, there are also several points worth elaborating. First, unlike the previous studies, we modeled the between‐study heterogeneity using the between‐study covariance matrix T instead of a fixed bias. According to Spiegelhalter et al., there are different assumptions on the between‐study heterogeneity, including equal but discounted, exchangeable, and biased.^32^ The equal but discounted assumption is the assumption of the power prior but it is impossible to model the between‐study heterogeneity based on this assumption. The exchangeable assumption assumes parameters from different studies are from the same distribution, which is the assumption of the meta‐analytic‐predictive and the commensurate prior approach. We used the exchangeable assumption to model the between‐study heterogeneity in this study. The biased assumption assumes there is a systematic bias between the current parameter and the historical parameter, which was widely used in previous studies.^1^, ^2^, ^3^, ^21^, ^33^ Compared to the exchangeable assumption used in this study, the biased assumption is more realistic because the historical data is often available when researchers are choosing the historical borrowing method. Thus, the bias between the historical parameter and the current parameter is assumed to be fixed. However, it is hard to show the benefit of a historical borrowing method in that it is impossible to gain power with type I error rate controlled given the historical data.^30^ The advantage of the exchangeable assumption is that it is more general, that is, not limited to a specific historical data set, because the operating characteristics are derived by averaging over different simulated historical data sets, and it is more convenient to compare historical borrowing methods in terms of type I error rate and power based on the assumption. Yet researchers may need to be wary of the historical control that violates the exchangeable assumption, that is, prior‐data conflict. In practice, the exchangeable assumption is more appropriate if the evaluation of the methods is supposed to be generalized, while the biased assumption is preferred given the historical data. Second, we only considered equal sample sizes for the historical control arm and the current trial in the simulation. In practice, it is likely that the two trials have different sample sizes, so further research is of interest to evaluate how the sample size of the historical control arm could affect the operating characteristics.

In addition to its implementation in longitudinal data analysis, the MPP could also be used to reduce the sample size in a new trial with a historical control arm available. However, the simulation results imply that the power gain is limited with the type I error rate controlled at the nominal 5% level. Therefore, the practical value of the MPP in sample size calculation may be limited, that is, the required sample size would only be slightly reduced.

The conditional MPP might be the only feasible choice to implement the MPP in the generalized linear mixed models (GLMM) because no closed‐form solution is available to integrate the random effects out of the historical likelihood in the GLMM. However, the sampler has difficulty in posterior sampling in terms of computational time and number of iterations required to achieve convergence in the implementation of this approach and the probable reason is that a large number of historical random effects need to be sampled with uncertain amount of historical borrowing. Moreover, the potential bimodality of the power parameter is an intrinsic property of the conditional MPP, which makes the method hard to sample and interpret. Finally, the results of the conditional MPP in LMMs have shown that the approach has borrowed extra historical information even with a relatively low between‐study heterogeneity, which leads to an inflated type I error rate. Based on its unfavorable characteristics including low computational efficiency, bimodality of the power parameter, and excessive borrowing of historical information, the conditional MPP is not recommended to incorporate historical information in longitudinal data analysis. We are currently exploring what dynamic borrowing methods are best for the GLMM.

Furthermore, we implemented the MPP assuming the same set of covariates and same G matrix across studies, which was the initial assumption of the MPP. If the true underlying parameters in two trials do differ, the power parameter estimate will be low and the historical information will be greatly downweighted. In practice, it is also possible to implement the MPP with different sets of covariates or G matrices in different trials using the partial borrowing power prior,^3^ which only borrows the shared parameters in the historical control arm and the current trial.

The ADCS data used in our study are ideal candidates to illustrate dynamic historical borrowing methods because they have (a) the same study design, (b) the same control arm treatment, (c) the same inclusion criteria, and (d) comparable baseline characteristics. Besides, they are consecutive clinical trials conducted by the same research group and even published in the same journal.^23^, ^24^ It then became natural to consider borrowing the historical control arm when analyzing the current data given the comparability between the historical and current data. Similarly, researchers who are interested in incorporating historical control arm in the current analysis using historical borrowing methods should also evaluate the compatibility between historical and current data beforehand. The criteria proposed by Pocock are common choices to accomplish the evaluation.^28^, ^34^ The results of the real‐data analysis in this study were in accordance with those in the simulation study, and the conditional MPP borrowed the most among the dynamic borrowing methods and the commensurate prior borrowed the least. None of the dynamic borrowing methods find clear evidence that DHA supplementation slows the rate of cognitive decline in patients with Alzheimer's disease. Although this conclusion is in line with the original publication of the ADC027 trial,^24^ the results of the dynamic borrowing methods provide a bit more precision on the potential treatment effect (reduce the posterior *SD* by 3.9%–19.6%).

In conclusion, the conditional MPP is not recommended for incorporating the historical controls in clinical trials with longitudinal outcomes because it tends to borrow an excessive amount of the historical data even the between‐study heterogeneity is relatively high. On the contrary, the marginal MPP can yield more statistical power with the type I error rate close to 5%. Thus, it is sensible to further implement the marginal MPP in clinical trials with longitudinal normal responses. For instance, we may implement the marginal MPP to studies with multiple historical clinical trials because researchers often conduct clinical trials on the same disease with different investigational arms, such as ADAS for Alzheimer's disease used in this study and ACTG for HIV/AIDS.^35^ The MPP has been implemented in incorporating multiple historical trials with a single endpoint,^11^, ^21^ it is also worthwhile to extend the marginal MPP when multiple historical control arms with longitudinal outcomes are available in future studies. Furthermore, it is noteworthy that the commensurate prior outperforms the MPP in historical borrowing for highly heterogeneous data sets. Therefore the commensurate prior can be a viable choice to incorporate historical controls and prevent the data‐prior conflict.

## Supporting information


**Appendix S1**: Supporting Information.

## Data Availability

The example data in the article can be requested from the ADCS data management team. Data used in the study were obtained from the University of California, San Diego Alzheimer's Disease Cooperative Study, https://www.adcs.org/.
