# Supplementary material for: Incorporating historical controls in clinical trials with longitudinal outcomes using the modified power prior
Source: Pharm Stat. 2022 Feb 6;21(5):818–34. doi: 10.1002/pst.2195 (PMC9356117; doi:10.1002/pst.2195)
Supplement: Supplementary file 1 — Appendix S1: Supporting Information. [file PST-21-818-s001.pdf]

Supplementary material for "Incorporating historical controls in clinical trials with longitudinal outcomes using the modified power prior"

Hongchao Qi[1,2]*, Dimitris Rizopoulos[1,2], Emmanuel Lesaffre[3], Joost van Rosmalen[1,2]

[1] Department of Biostatistics, Erasmus University Medical Center, Rotterdam, The Netherlands

[2] Department of Epidemiology, Erasmus University Medical Center, Rotterdam, The Netherlands

[3] I-Biostat, KU-Leuven, Leuven, Belgium

In the supplementary material, we present a theoretical comparison between the conditional MPP and the marginal MPP in Section 1, and the sampling method for the posterior of the conditional MPP and the marginal MPP in Section 2 and Section 3. The results of the simulation study are presented in Section 4. The results of the sensitivity analysis for the conditional MPP and the marginal MPP with a more skeptical $\text{Beta}(1, 2)$ prior for the power parameter are provided in Section 5. The results of the sensitivity analysis for the LKJ prior in the real data analysis are presented in Section 6. Graphical convergence diagnostics for the motivating data analysis are presented in Section 7. Finally, information on the R and Stan syntax files for the simulation study is presented in Section 8.

*Correspondence to: Hongchao Qi, Department of Biostatistics, Erasmus MC, P.O. Box 2040, 3000CA Rotterdam, the Netherlands. E-mail: h.qi@erasmusmc.nl.

1 The comparison between the conditional MPP and the marginal MPP

In this section, a theoretical comparison between the conditional MPP and the marginal MPP is presented. Suppose that the LMMs for the current study and the historical control arm are as follows

$$\boldsymbol{y}_i = \boldsymbol{X}_i\,\boldsymbol{\beta}_C + d_i\,\beta_T + \boldsymbol{Z}_i\,\boldsymbol{b}_i + \boldsymbol{\epsilon}_i, \tag{S1}$$

and

$$\boldsymbol{y}_{0i'} = \boldsymbol{X}_{0i'}\,\boldsymbol{\beta}_C + \boldsymbol{Z}_{0i'}\,\boldsymbol{b}_{0i'} + \boldsymbol{\epsilon}_{0i'}. \tag{S2}$$

The posterior of the model parameters with the conditional MPP and the marginal MPP can be derived based on the above models.

The posterior of the model parameters in the conditional MPP is

$$\begin{aligned}
&p(\boldsymbol{\beta}_C, \beta_T, \boldsymbol{G}, \sigma^2, \boldsymbol{b}, \boldsymbol{b}_0, \alpha \mid \boldsymbol{y}, \boldsymbol{y}_0) = \\
&\left[\prod_{i=1}^{n} p(\boldsymbol{y}_i \mid \boldsymbol{\beta}_C, \beta_T, \boldsymbol{b}_i, \sigma^2)\, p(\boldsymbol{b}_i \mid \boldsymbol{G})\right] p(\beta_T) \frac{\left[\prod_{i'=1}^{n_0} p(\boldsymbol{y}_{0i'} \mid \boldsymbol{\beta}_C, \boldsymbol{b}_{0i'}, \sigma^2)^{\alpha}\, p(\boldsymbol{b}_{0i'} \mid \boldsymbol{G})\right] p(\boldsymbol{\theta})}{\int\int \left[\prod_{i'=1}^{n_0} p(\boldsymbol{y}_{0i'} \mid \boldsymbol{\beta}_C, \boldsymbol{b}_{0i'}, \sigma^2)^{\alpha}\, p(\boldsymbol{b}_{0i'} \mid \boldsymbol{G})\right] p(\boldsymbol{\theta}) d\boldsymbol{b}_0 d\boldsymbol{\theta}} p(\alpha) = \\
&\left[\prod_{i=1}^{n} \frac{\exp(-\frac{1}{2}\boldsymbol{A}_i^T \boldsymbol{R}_i^{-1} \boldsymbol{A}_i)}{\sqrt{(2\pi)^{m_i} \mid \boldsymbol{R}_i \mid}} \frac{\exp(-\frac{1}{2}\boldsymbol{b}_i^T \boldsymbol{G}^{-1} \boldsymbol{b}_i)}{\sqrt{(2\pi)^{q} \mid \boldsymbol{G} \mid}}\right] p(\beta_T) \frac{\left[\prod_{i'=1}^{n_0} \left(\frac{\exp(-\frac{1}{2}\boldsymbol{A}_{0i'}^T \boldsymbol{R}_{0i'}^{-1} \boldsymbol{A}_{0i'})}{\sqrt{(2\pi)^{m_{0i'}} \mid \boldsymbol{R}_{0i'} \mid}}\right)^{\alpha} \frac{\exp(-\frac{1}{2}\boldsymbol{b}_{0i'}^T \boldsymbol{G}^{-1} \boldsymbol{b}_{0i'})}{\sqrt{(2\pi)^{q} \mid \boldsymbol{G} \mid}}\right] p(\boldsymbol{\theta})}{\int \left[\prod_{i'=1}^{n_0} \int \left(\frac{\exp(-\frac{1}{2}\boldsymbol{A}_{0i'}^T \boldsymbol{R}_{0i'}^{-1} \boldsymbol{A}_{0i'})}{\sqrt{(2\pi)^{m_{0i'}} \mid \boldsymbol{R}_{0i'} \mid}}\right)^{\alpha} \frac{\exp(-\frac{1}{2}\boldsymbol{b}_{0i'}^T \boldsymbol{G}^{-1} \boldsymbol{b}_{0i'})}{\sqrt{(2\pi)^{q} \mid \boldsymbol{G} \mid}} d\boldsymbol{b}_{0i'}\right] p(\boldsymbol{\theta}) d\boldsymbol{\theta}} p(\alpha),
\end{aligned} \tag{S3}$$

where $\boldsymbol{A}_i = \boldsymbol{y}_i - \boldsymbol{X}_i\boldsymbol{\beta}_C - d_i\beta_T - \boldsymbol{Z}_i\boldsymbol{b}_i$, $\boldsymbol{A}_{0i'} = \boldsymbol{y}_{0i'} - \boldsymbol{X}_{0i'}\boldsymbol{\beta}_C - \boldsymbol{Z}_{0i'}\boldsymbol{b}_{0i'}$ and $\boldsymbol{\theta} = (\boldsymbol{\beta}_C, \boldsymbol{G}, \sigma^2)$.

The posterior in the marginal MPP is obtained based on the marginal likelihood, which is given by

$$p(\boldsymbol{\beta}_C, \beta_T, \boldsymbol{G}, \sigma^2, \alpha \mid \boldsymbol{y}, \boldsymbol{y}_0) =$$

$$\left[\prod_{i=1}^{n} \int p(\boldsymbol{y}_i \mid \boldsymbol{\beta}_C, \beta_T, \boldsymbol{b}_i, \sigma^2)\, p(\boldsymbol{b}_i \mid \boldsymbol{G}) d\boldsymbol{b}_i\right] p(\beta_T) \frac{\left[\prod_{i'=1}^{n_0} \int p(\boldsymbol{y}_{0i'} \mid \boldsymbol{\beta}_C, \boldsymbol{b}_{0i'}, \sigma^2)\, p(\boldsymbol{b}_{0i'} \mid \boldsymbol{G}) d\boldsymbol{b}_{0i'}\right]^{\alpha} p(\boldsymbol{\theta})}{\int \left[\prod_{i'=1}^{n_0} \int p(\boldsymbol{y}_{0i'} \mid \boldsymbol{\beta}_C, \boldsymbol{b}_{0i'}, \sigma^2)\, p(\boldsymbol{b}_{0i'} \mid \boldsymbol{G}) d\boldsymbol{b}_{0i'}\right]^{\alpha} p(\boldsymbol{\theta}) d\boldsymbol{\theta}} p(\alpha) =$$

$$\left[\prod_{i=1}^{n} \left(\int \frac{\exp(-\frac{1}{2}\boldsymbol{A}_i^T \boldsymbol{R}_i^{-1} \boldsymbol{A}_i)}{\sqrt{(2\pi)^{m_i} \mid \boldsymbol{R}_i \mid}} \frac{\exp(-\frac{1}{2}\boldsymbol{b}_i^T \boldsymbol{G}^{-1} \boldsymbol{b}_i)}{\sqrt{(2\pi)^{q} \mid \boldsymbol{G} \mid}} d\boldsymbol{b}_i\right)\right] p(\beta_T) \frac{\left[\prod_{i'=1}^{n_0} \left(\int \frac{\exp(-\frac{1}{2}\boldsymbol{A}_{0i'}^T \boldsymbol{R}_{0i'}^{-1} \boldsymbol{A}_{0i'})}{\sqrt{(2\pi)^{m_{0i'}} \mid \boldsymbol{R}_{0i'} \mid}} \frac{\exp(-\frac{1}{2}\boldsymbol{b}_{0i'}^T \boldsymbol{G}^{-1} \boldsymbol{b}_{0i'})}{\sqrt{(2\pi)^{q} \mid \boldsymbol{G} \mid}} d\boldsymbol{b}_{0i'}\right)^{\alpha}\right] p(\boldsymbol{\theta})}{\int \left[\prod_{i'=1}^{n_0} \left(\int \frac{\exp(-\frac{1}{2}\boldsymbol{A}_{0i'}^T \boldsymbol{R}_{0i'}^{-1} \boldsymbol{A}_{0i'})}{\sqrt{(2\pi)^{m_{0i'}} \mid \boldsymbol{R}_{0i'} \mid}} \frac{\exp(-\frac{1}{2}\boldsymbol{b}_{0i'}^T \boldsymbol{G}^{-1} \boldsymbol{b}_{0i'})}{\sqrt{(2\pi)^{q} \mid \boldsymbol{G} \mid}} d\boldsymbol{b}_{0i'}\right)^{\alpha}\right] p(\boldsymbol{\theta}) d\boldsymbol{\theta}} p(\alpha) =$$

$$\left[\prod_{i=1}^{n} \frac{\exp(-\frac{1}{2}\boldsymbol{B}_i^T \boldsymbol{V}_i^{-1} \boldsymbol{B}_i)}{\sqrt{(2\pi)^{m_i} \mid \boldsymbol{V}_i \mid}}\right] p(\beta_T) \frac{\left[\prod_{i'=1}^{n_0} \frac{\exp(-\frac{1}{2}\alpha \boldsymbol{B}_{0i'}^T (\boldsymbol{Z}_{0i'} \boldsymbol{G} \boldsymbol{Z}_{0i'}^T + \boldsymbol{R}_{0i'})^{-1} \boldsymbol{B}_{0i'})}{(\sqrt{(2\pi)^{m_{0i'}} \mid \boldsymbol{Z}_{0i'} \boldsymbol{G} \boldsymbol{Z}_{0i'}^T + \boldsymbol{R}_{0i'} \mid})^{\alpha}}\right] p(\boldsymbol{\theta})}{\int \left[\prod_{i'=1}^{n_0} \frac{\exp(-\frac{1}{2}\alpha \boldsymbol{B}_{0i'}^T (\boldsymbol{Z}_{0i'} \boldsymbol{G} \boldsymbol{Z}_{0i'}^T + \boldsymbol{R}_{0i'})^{-1} \boldsymbol{B}_{0i'})}{(\sqrt{(2\pi)^{m_{0i'}} \mid \boldsymbol{Z}_{0i'} \boldsymbol{G} \boldsymbol{Z}_{0i'}^T + \boldsymbol{R}_{0i'} \mid})^{\alpha}}\right] p(\boldsymbol{\theta})\, d\boldsymbol{\theta}} p(\alpha), \quad \text{(S4)}$$

where $\boldsymbol{V}_i = \boldsymbol{Z}_i \boldsymbol{G} \boldsymbol{Z}_i^T + \boldsymbol{R}_i$, $\boldsymbol{B}_i = \boldsymbol{y}_i - \boldsymbol{X}_i \boldsymbol{\beta}_C - d_i \beta_T$ and $\boldsymbol{B}_{0i'} = \boldsymbol{y}_{0i'} - \boldsymbol{X}_{0i'} \boldsymbol{\beta}_C$. If we rescale the covariance matrix, $\boldsymbol{Z}_{0i'} \boldsymbol{G} \boldsymbol{Z}_{0i'}^T + \boldsymbol{R}_{0i'}$, with the power parameter α, the posterior in the marginal MPP then becomes

$$\left[\prod_{i=1}^{n} \frac{\exp(-\frac{1}{2}\boldsymbol{B}_i^T \boldsymbol{V}_i^{-1} \boldsymbol{B}_i)}{\sqrt{(2\pi)^{m_i} \mid \boldsymbol{V}_i \mid}}\right] p(\beta_T) \frac{\left[\prod_{i'=1}^{n_0} \frac{\sqrt{(2\pi)^{m_{0i'}} \mid \boldsymbol{Z}_{0i'} \frac{\boldsymbol{G}}{\alpha} \boldsymbol{Z}_{0i'}^T + \frac{\boldsymbol{R}_{0i'}}{\alpha} \mid}}{(\sqrt{(2\pi)^{m_{0i'}} \mid \boldsymbol{Z}_{0i'} \boldsymbol{G} \boldsymbol{Z}_{0i'}^T + \boldsymbol{R}_{0i'} \mid})^{\alpha}} \frac{\exp(-\frac{1}{2}\boldsymbol{B}_{0i'}^T (\boldsymbol{Z}_{0i'} \frac{\boldsymbol{G}}{\alpha} \boldsymbol{Z}_{0i'}^T + \frac{\boldsymbol{R}_{0i'}}{\alpha})^{-1} \boldsymbol{B}_{0i'})}{\sqrt{(2\pi)^{m_{0i'}} \mid \boldsymbol{Z}_{0i'} \frac{\boldsymbol{G}}{\alpha} \boldsymbol{Z}_{0i'}^T + \frac{\boldsymbol{R}_{0i'}}{\alpha} \mid}}\right] p(\boldsymbol{\theta})}{\int \left[\prod_{i'=1}^{n_0} \frac{\sqrt{(2\pi)^{m_{0i'}} \mid \boldsymbol{Z}_{0i'} \frac{\boldsymbol{G}}{\alpha} \boldsymbol{Z}_{0i'}^T + \frac{\boldsymbol{R}_{0i'}}{\alpha} \mid}}{(\sqrt{(2\pi)^{m_{0i'}} \mid \boldsymbol{Z}_{0i'} \boldsymbol{G} \boldsymbol{Z}_{0i'}^T + \boldsymbol{R}_{0i'} \mid})^{\alpha}} \frac{\exp(-\frac{1}{2}\boldsymbol{B}_{0i'}^T (\boldsymbol{Z}_{0i'} \frac{\boldsymbol{G}}{\alpha} \boldsymbol{Z}_{0i'}^T + \frac{\boldsymbol{R}_{0i'}}{\alpha})^{-1} \boldsymbol{B}_{0i'})}{\sqrt{(2\pi)^{m_{0i'}} \mid \boldsymbol{Z}_{0i'} \frac{\boldsymbol{G}}{\alpha} \boldsymbol{Z}_{0i'}^T + \frac{\boldsymbol{R}_{0i'}}{\alpha} \mid}}\right] p(\boldsymbol{\theta})\, d\boldsymbol{\theta}} p(\alpha) =$$

$$\left[\prod_{i=1}^{n} \frac{\exp(-\frac{1}{2}\boldsymbol{B}_i^T \boldsymbol{V}_i^{-1} \boldsymbol{B}_i)}{\sqrt{(2\pi)^{m_i} \mid \boldsymbol{V}_i \mid}}\right] p(\beta_T) \frac{\left[\prod_{i'=1}^{n_0} \mid \boldsymbol{Z}_{0i'} \boldsymbol{G} \boldsymbol{Z}_{0i'}^T + \boldsymbol{R}_{0i'} \mid^{\frac{(1-\alpha)}{2}} \frac{\exp(-\frac{1}{2}\boldsymbol{B}_{0i'}^T (\boldsymbol{Z}_{0i'} \frac{\boldsymbol{G}}{\alpha} \boldsymbol{Z}_{0i'}^T + \frac{\boldsymbol{R}_{0i'}}{\alpha})^{-1} \boldsymbol{B}_{0i'})}{\sqrt{(2\pi)^{m_{0i'}} \mid \boldsymbol{Z}_{0i'} \frac{\boldsymbol{G}}{\alpha} \boldsymbol{Z}_{0i'}^T + \frac{\boldsymbol{R}_{0i'}}{\alpha} \mid}}\right] p(\boldsymbol{\theta})}{\int \left[\prod_{i'=1}^{n_0} \mid \boldsymbol{Z}_{0i'} \boldsymbol{G} \boldsymbol{Z}_{0i'}^T + \boldsymbol{R}_{0i'} \mid^{\frac{(1-\alpha)}{2}} \frac{\exp(-\frac{1}{2}\boldsymbol{B}_{0i'}^T (\boldsymbol{Z}_{0i'} \frac{\boldsymbol{G}}{\alpha} \boldsymbol{Z}_{0i'}^T + \frac{\boldsymbol{R}_{0i'}}{\alpha})^{-1} \boldsymbol{B}_{0i'})}{\sqrt{(2\pi)^{m_{0i'}} \mid \boldsymbol{Z}_{0i'} \frac{\boldsymbol{G}}{\alpha} \boldsymbol{Z}_{0i'}^T + \frac{\boldsymbol{R}_{0i'}}{\alpha} \mid}}\right] p(\boldsymbol{\theta})\, d\boldsymbol{\theta}} p(\alpha) \quad \text{(S5)}$$

To our knowledge, the marginal posterior of α does not have a closed form in the conditional MPP or the marginal MPP.

To help identify the difference between the conditional MPP and the marginal MPP, we first derive the marginal posterior of $\boldsymbol{\beta}_C$, β_T, $\boldsymbol{G}$, σ^2, and α by integrating out the random effects ($\boldsymbol{b}$, $\boldsymbol{b}_0$) in the conditional MPP, which is given by

$$p(\boldsymbol{\beta}_C, \beta_T, \boldsymbol{G}, \sigma^2, \alpha \mid \boldsymbol{y}, \boldsymbol{y}_0) =$$

$$\left[\prod_{i=1}^{n} \int \frac{\exp(-\frac{1}{2}\boldsymbol{A}_i^T \boldsymbol{R}_i^{-1} \boldsymbol{A}_i)}{\sqrt{(2\pi)^{m_i} \mid \boldsymbol{R}_i \mid}} \frac{\exp(-\frac{1}{2}\boldsymbol{b}_i^T \boldsymbol{G}^{-1} \boldsymbol{b}_i)}{\sqrt{(2\pi)^q \mid \boldsymbol{G} \mid}} d\boldsymbol{b}_i\right] p(\beta_T) \frac{\left[\prod_{i'=1}^{n_0} \int \frac{\exp(-\frac{1}{2}\alpha \boldsymbol{A}_{0i'}^T \boldsymbol{R}_{0i'}^{-1} \boldsymbol{A}_{0i'})}{(\sqrt{(2\pi)^{m_{0i'}} \mid \boldsymbol{R}_{0i'} \mid})^\alpha} \frac{\exp(-\frac{1}{2}\boldsymbol{b}_{0i'}^T \boldsymbol{G}^{-1} \boldsymbol{b}_{0i'})}{\sqrt{(2\pi)^q \mid \boldsymbol{G} \mid}} d\boldsymbol{b}_{0i'}\right] p(\boldsymbol{\theta})}{\int \left[\prod_{i'=1}^{n_0} \int \frac{\exp(-\frac{1}{2}\alpha \boldsymbol{A}_{0i'}^T \boldsymbol{R}_{0i'}^{-1} \boldsymbol{A}_{0i'})}{(\sqrt{(2\pi)^{m_{0i'}} \mid \boldsymbol{R}_{0i'} \mid})^\alpha} \frac{\exp(-\frac{1}{2}\boldsymbol{b}_{0i'}^T \boldsymbol{G}^{-1} \boldsymbol{b}_{0i'})}{\sqrt{(2\pi)^q \mid \boldsymbol{G} \mid}} d\boldsymbol{b}_{0i'}\right] p(\boldsymbol{\theta}) d\boldsymbol{\theta}} p(\alpha) =$$

$$\left[\prod_{i=1}^{n} \frac{\exp(-\frac{1}{2}\boldsymbol{B}_i^T \boldsymbol{V}_i^{-1} \boldsymbol{B}_i)}{\sqrt{(2\pi)^{m_i} \mid \boldsymbol{V}_i \mid}}\right] p(\beta_T) \frac{\left[\prod_{i'=1}^{n_0} \frac{\sqrt{(2\pi)^{m_{0i'}} \mid \frac{\boldsymbol{R}_{0i'}}{\alpha} \mid}}{(\sqrt{(2\pi)^{m_{0i'}} \mid \boldsymbol{R}_{0i'} \mid})^\alpha} \int \frac{\exp(-\frac{1}{2}\boldsymbol{A}_{0i'}^T (\frac{\boldsymbol{R}_{0i'}}{\alpha})^{-1} \boldsymbol{A}_{0i'})}{\sqrt{(2\pi)^{m_{0i'}} \mid \frac{\boldsymbol{R}_{0i'}}{\alpha} \mid}} \frac{\exp(-\frac{1}{2}\boldsymbol{b}_{0i'}^T \boldsymbol{G}^{-1} \boldsymbol{b}_{0i'})}{\sqrt{(2\pi)^q \mid \boldsymbol{G} \mid}} d\boldsymbol{b}_{0i'}\right] p(\boldsymbol{\theta})}{\int \left[\prod_{i'=1}^{n_0} \frac{\sqrt{(2\pi)^{m_{0i'}} \mid \frac{\boldsymbol{R}_{0i'}}{\alpha} \mid}}{(\sqrt{(2\pi)^{m_{0i'}} \mid \boldsymbol{R}_{0i'} \mid})^\alpha} \int \frac{\exp(-\frac{1}{2}\boldsymbol{A}_{0i'}^T (\frac{\boldsymbol{R}_{0i'}}{\alpha})^{-1} \boldsymbol{A}_{0i'})}{\sqrt{(2\pi)^{m_{0i'}} \mid \frac{\boldsymbol{R}_{0i'}}{\alpha} \mid}} \frac{\exp(-\frac{1}{2}\boldsymbol{b}_{0i'}^T \boldsymbol{G}^{-1} \boldsymbol{b}_{0i'})}{\sqrt{(2\pi)^q \mid \boldsymbol{G} \mid}} d\boldsymbol{b}_{0i'}\right] p(\boldsymbol{\theta}) d\boldsymbol{\theta}} p(\alpha) =$$

$$\left[\prod_{i=1}^{n} \frac{\exp(-\frac{1}{2}\boldsymbol{B}_i^T \boldsymbol{V}_i^{-1} \boldsymbol{B}_i)}{\sqrt{(2\pi)^{m_i} \mid \boldsymbol{V}_i \mid}}\right] p(\beta_T) \frac{\left[\prod_{i'=1}^{n_0} \mid \boldsymbol{R}_{0i'} \mid^{\frac{(1-\alpha)}{2}} \frac{\exp(-\frac{1}{2}\boldsymbol{B}_{0i'}^T (\boldsymbol{Z}_{0i'} \boldsymbol{G} \boldsymbol{Z}_{0i'}^T + \frac{\boldsymbol{R}_{0i'}}{\alpha})^{-1} \boldsymbol{B}_{0i'})}{\sqrt{(2\pi)^{m_{0i'}} \mid \boldsymbol{Z}_{0i'} \boldsymbol{G} \boldsymbol{Z}_{0i'}^T + \frac{\boldsymbol{R}_{0i'}}{\alpha} \mid}}\right] p(\boldsymbol{\theta})}{\int \left[\prod_{i'=1}^{n_0} \mid \boldsymbol{R}_{0i'} \mid^{\frac{(1-\alpha)}{2}} \frac{\exp(-\frac{1}{2}\boldsymbol{B}_{0i'}^T (\boldsymbol{Z}_{0i'} \boldsymbol{G} \boldsymbol{Z}_{0i'}^T + \frac{\boldsymbol{R}_{0i'}}{\alpha})^{-1} \boldsymbol{B}_{0i'})}{\sqrt{(2\pi)^{m_{0i'}} \mid \boldsymbol{Z}_{0i'} \boldsymbol{G} \boldsymbol{Z}_{0i'}^T + \frac{\boldsymbol{R}_{0i'}}{\alpha} \mid}}\right] p(\boldsymbol{\theta}) d\boldsymbol{\theta}} p(\alpha). \quad \text{(S6)}$$

Equation (S5) and Equation (S6) differ in terms of (a) the compensate term in front of the multivariate normal distribution, and (b) the covariance matrix of the multivariate normal distribution.

To simplify the comparison between the conditional MPP and the marginal MPP, we can assume $\boldsymbol{G}$ and σ^2 to be known so that the compensate terms in front of the multivariate normal distribution in Equation (S5) and Equation (S6) can be canceled out. Based on the above formulations, the conditional MPP only inflates the variance of the multivariate normal distribution of $\boldsymbol{y}_{0i'}$ with a weight of $\frac{1}{\alpha}$ for the error variance, but the marginal MPP inflates the variance of the multivariate normal distribution with the weight $\frac{1}{\alpha}$ for both variances of random effects and the error variance. Thus the conditional MPP tends to borrow more historical information given the same power value.

In conclusion, we have found in the above comparison that the closed forms of the power parameter posterior in both approaches are not available, and interpretations of the same power value in both approaches are different because the same value leads to different amount of borrowing.

2 The sampling for the posterior of the MPP

The sampling for the posterior of the MPP consists of two main steps, including (1) the calculation of the scaling constants corresponding to different fixed power values, and (2) the sampling from the posterior distribution based on the scaling constants calculated in the first step. The algorithm is adapted from a similar algorithm proposed in our previous study [1].

2.1 *Step 1*: Calculation of the scaling constant

In this step, a path sampling algorithm is adopted to calculate the scaling constant. In the algorithm, the logarithm of $C(\alpha)$ is equal to the expected log-likelihood of the historical data as a function of parameters that are sampled from the power prior, i.e.,

$$\log(C(\alpha)) = \int_{\alpha=0}^{\alpha} E_{p(\boldsymbol{\theta},\boldsymbol{b}_0|\alpha,\boldsymbol{y}_0)} \log L(\boldsymbol{\theta}, \boldsymbol{b}_0 \mid \boldsymbol{y}_0) d\alpha$$

for the conditional MPP, and

$$\log(C(\alpha)) = \int_{\alpha=0}^{\alpha} E_{p(\boldsymbol{\theta}|\alpha,\boldsymbol{y}_0)} \log L(\boldsymbol{\theta} \mid \boldsymbol{y}_0) d\alpha$$

for the marginal MPP, where $\boldsymbol{\theta}$ is the model parameters such as regression coefficients, covariance matrix and error variance, and $\boldsymbol{b}_0$ is the historical random effects. The details of the algorithm are as follows.

1. Choose Δ_α, the increase in α per iteration and n_{iter}, the number of HMC samples per iteration. Initialize $\alpha = 0$, and initialize the model parameters using a draw from the prior $p(\boldsymbol{\theta})$.

2. Repeat the following until $\alpha \geq 1$:

   (a) Increase the value of α by Δ_α;

   (b) Sample n_{iter} HMC iterations from the power prior distribution with fixed power parameter α, which is

   $$\frac{L(\boldsymbol{\theta}, \boldsymbol{b}_0 \mid \boldsymbol{y}_0)^\alpha \, p(\boldsymbol{b}_0 \mid \boldsymbol{\theta}) \, p(\boldsymbol{\theta})}{\int\int L(\boldsymbol{\theta}, \boldsymbol{b}_0 \mid \boldsymbol{y}_0)^\alpha \, p(\boldsymbol{b}_0 \mid \boldsymbol{\theta}) \, p(\boldsymbol{\theta}) d\boldsymbol{b}_0 d\boldsymbol{\theta}}$$

   for the conditional MPP or

   $$\frac{\left[\int L(\boldsymbol{\theta}, \boldsymbol{b}_0 \mid \boldsymbol{y}_0) \, p(\boldsymbol{b}_0 \mid \boldsymbol{\theta}) d\boldsymbol{b}_0\right]^\alpha \, p(\boldsymbol{\theta})}{\int \left[\int L(\boldsymbol{\theta}, \boldsymbol{b}_0 \mid \boldsymbol{y}_0) \, p(\boldsymbol{b}_0 \mid \boldsymbol{\theta}) d\boldsymbol{b}_0\right]^\alpha \, p(\boldsymbol{\theta}) d\boldsymbol{\theta}}$$

   for the marginal MPP;

   (c) Calculate the average log-likelihood of the historical data, i.e., $E(\log L(\boldsymbol{\theta}, \boldsymbol{b}_0 \mid \boldsymbol{y}_0))$ for the conditional MPP and $E(\log L(\boldsymbol{\theta} \mid \boldsymbol{y}_0))$ for the marginal MPP, using all the parameter sets sampled for the current value of α.

3. Calculate the cumulative sum of the average log-likelihood values that were calculated in the last step, as a function of α.

4. $C(\alpha)$ is now proportional to the exponential of the cumulative sum calculated in the previous step.

2.2 *Step 2*: Sampling from the posterior distribution

In the second step, model parameters and the power parameter are sampled using HMC from

$$p(\boldsymbol{\theta}, \boldsymbol{b}, \boldsymbol{b}_0, \alpha \mid \boldsymbol{y}, \boldsymbol{y}_0) \propto L(\boldsymbol{\theta}, \boldsymbol{b} \mid \boldsymbol{y})\, p(\boldsymbol{b} \mid \boldsymbol{\theta}) \frac{L(\boldsymbol{\theta}, \boldsymbol{b}_0 \mid \boldsymbol{y}_0)^{\alpha}\, p(\boldsymbol{b}_0 \mid \boldsymbol{\theta})\, p(\boldsymbol{\theta})}{C(\alpha)} p(\alpha)$$

for the conditional MPP or

$$p(\boldsymbol{\theta}, \alpha \mid \boldsymbol{y}, \boldsymbol{y}_0) \propto \int L(\boldsymbol{\theta}, \boldsymbol{b} \mid \boldsymbol{y})\, p(\boldsymbol{b} \mid \boldsymbol{\theta}) d\boldsymbol{b} \frac{\left[\int L(\boldsymbol{\theta}, \boldsymbol{b}_0 \mid \boldsymbol{y}_0)\, p(\boldsymbol{b}_0 \mid \boldsymbol{\theta}) d\boldsymbol{b}_0\right]^{\alpha} p(\boldsymbol{\theta})}{C(\alpha)} p(\alpha)$$

for the marginal MPP, where $\boldsymbol{b}$ is the current random effects and $C(\alpha)$ is calculated with linear interpolation using the grid of fixed power values and their corresponding values of $\log(C(\alpha))$ obtained from *Step 1*.

3 Sampling the posterior in the conditional MPP

In the second step of the above algorithm, the efficiency of the sampler for the conditional MPP is relatively low due to a large number of historical random effects to be sampled. In linear mixed models, we can avoid sampling the historical random effects by integrating them out to improve the computational efficiency. The marginal posterior of $\boldsymbol{\beta}_C$, β_T, $\boldsymbol{b}$, $\boldsymbol{G}$, σ^2, and α is as follows.

$$\begin{aligned} p(\boldsymbol{\beta}_C, \beta_T, \boldsymbol{b}, \boldsymbol{G}, \sigma^2, \alpha \mid \boldsymbol{y}_0, \boldsymbol{y}) \propto & \left[\prod_{i=1}^{n} \frac{\exp(-\frac{1}{2}\boldsymbol{A}_i^T \boldsymbol{R}_i^{-1} \boldsymbol{A}_i)}{\sqrt{(2\pi)^{m_i} \mid \boldsymbol{R}_i \mid}} \frac{\exp(-\frac{1}{2}\boldsymbol{b}_i^T \boldsymbol{G}^{-1} \boldsymbol{b}_i)}{\sqrt{(2\pi)^{q} \mid \boldsymbol{G} \mid}}\right] p(\beta_T) \times \\ & \frac{\left[\prod_{i'=1}^{n_0} (2\pi)^{\frac{m_{0i'}(1-\alpha)}{2}} \alpha^{\frac{-m_{0i'}}{2}} \mid \boldsymbol{R}_{0i'} \mid^{\frac{(1-\alpha)}{2}} \frac{\exp(-\frac{1}{2}\boldsymbol{B}_{0i'}^T (\boldsymbol{Z}_{0i'} \boldsymbol{G} \boldsymbol{Z}_{0i'}^T + \frac{\boldsymbol{R}_{0i'}}{\alpha})^{-1} \boldsymbol{B}_{0i'})}{\sqrt{(2\pi)^{m_{0i'}} \mid \boldsymbol{Z}_{0i'} \boldsymbol{G} \boldsymbol{Z}_{0i'}^T + \frac{\boldsymbol{R}_{0i'}}{\alpha} \mid}}\right] p(\boldsymbol{\theta})}{\int \left[\prod_{i'=1}^{n_0} \int \left(\frac{\exp(-\frac{1}{2}\boldsymbol{A}_{0i'}^T \boldsymbol{R}_{0i'}^{-1} \boldsymbol{A}_{0i'})}{\sqrt{(2\pi)^{m_{0i'}} \mid \boldsymbol{R}_{0i'} \mid}}\right)^{\alpha} \frac{\exp(-\frac{1}{2}\boldsymbol{b}_{0i'}^T \boldsymbol{G}^{-1} \boldsymbol{b}_{0i'})}{\sqrt{(2\pi)^{q} \mid \boldsymbol{G} \mid}} d\boldsymbol{b}_{0i'}\right] p(\boldsymbol{\theta}) d\boldsymbol{\theta}} p(\alpha), \end{aligned} \quad \text{(S7)}$$

where the scaling constant in the denominator is calculated in *Step 1*. According to preliminary simulations, the new sampler is more efficient than the sampler with random effects in terms of (a) computational time, and (b) number of iterations required to achieve convergence.

4 Results of the simulation study

In the following tables, we presented the results of the simulation including the type I error rate and the statistical power, the performance measures of effect estimation, and distributions of power parameters in the conditional MPP and the marginal MPP. In the tables, $\beta_2 = 0$ implies scenarios without treatment effect, while $\beta_2 = 0.36$ indicates scenarios with treatment effect.

Table S1: The type I error rate and statistical power (%) of the estimated treatment effect (Monte Carlo SE in parentheses) in the simulation study based on 500 simulated data sets

Method	Scenario for between-study heterogeneity						
	No	RI+Low	RI+Moderate	RI+High	RIS+Low	RIS+Moderate	RIS+High
$\beta_2 = 0$							
No borrowing	5.2 (1.0)	4.8 (1.0)	4.2 (0.9)	5.8 (1.0)	4.0 (0.9)	4.0 (0.9)	5.4 (1.0)
Conditional MPP	5.4 (1.0)	6.4 (1.1)	7.8 (1.2)	9.0 (1.3)	6.0 (1.1)	8.2 (1.2)	10.4 (1.4)
Marginal MPP	5.2 (1.0)	5.6 (1.0)	5.6 (1.0)	6.2 (1.1)	5.2 (1.0)	5.4 (1.0)	8.8 (1.3)
Commensurate prior	5.2 (1.0)	4.8 (1.0)	4.6 (0.9)	6.2 (1.1)	4.0 (0.9)	3.8 (0.9)	5.6 (1.0)
Pooling	5.4 (1.0)	7.2 (1.2)	15.2 (1.6)	26.6 (2.0)	7.2 (1.2)	26.2 (2.0)	42.2 (2.2)
$\beta_2 = 0.36$							
No borrowing	71.2 (2.0)	73.0 (2.0)	71.8 (2.0)	71.2 (2.0)	71.8 (2.0)	70.0 (2.0)	72.0 (2.0)
Conditional MPP	82.2 (1.7)	82.4 (1.7)	77.4 (1.9)	73.8 (2.0)	81.2 (1.7)	72.0 (2.0)	70.8 (2.0)
Marginal MPP	81.6 (1.7)	81.6 (1.7)	78.0 (1.9)	75.6 (1.9)	80.8 (1.8)	73.6 (2.0)	70.4 (2.0)
Commensurate prior	75.8 (1.9)	75.8 (1.9)	74.8 (1.9)	73.4 (2.0)	74.4 (2.0)	70.6 (2.0)	72.4 (2.0)
Pooling	82.4 (1.7)	83.6 (1.7)	75.6 (1.9)	70.6 (2.0)	81.6 (1.7)	69.8 (2.1)	66.0 (2.1)

Table S2: The average bias (Monte Carlo SE in parentheses) of the estimated treatment effect in the simulation study based on 500 simulated data sets

Method	Scenario for between-study heterogeneity						
	No	RI+Low	RI+Moderate	RI+High	RIS+Low	RIS+Moderate	RIS+High
$\beta_2 = 0$							
No borrowing	−0.006 (0.006)	−0.007 (0.006)	0.016 (0.006)	0.002 (0.006)	−0.004 (0.006)	0.005 (0.006)	0.005 (0.006)
Conditional MPP	−0.001 (0.006)	−0.002 (0.006)	0.016 (0.006)	0.003 (0.007)	−0.001 (0.006)	0.015 (0.007)	0.005 (0.007)
Marginal MPP	−0.001 (0.006)	−0.001 (0.006)	0.018 (0.006)	0.006 (0.006)	0.000 (0.006)	0.013 (0.006)	0.007 (0.007)
Commensurate prior	−0.005 (0.006)	−0.006 (0.006)	0.016 (0.006)	0.003 (0.006)	−0.005 (0.006)	0.005 (0.006)	0.004 (0.006)
Pooling	−0.005 (0.006)	−0.006 (0.006)	0.012 (0.008)	0.000 (0.010)	−0.004 (0.006)	0.004 (0.010)	0.011 (0.013)
$\beta_2 = 0.36$							
No borrowing	−0.002 (0.006)	0.006 (0.006)	0.001 (0.007)	0.003 (0.007)	0.002 (0.006)	−0.005 (0.006)	−0.001 (0.006)
Conditional MPP	−0.002 (0.006)	0.013 (0.006)	0.004 (0.007)	0.003 (0.007)	0.006 (0.006)	0.000 (0.007)	0.000 (0.007)
Marginal MPP	0.000 (0.006)	0.013 (0.006)	0.006 (0.006)	0.005 (0.007)	0.007 (0.006)	0.001 (0.007)	0.000 (0.006)
Commensurate prior	−0.001 (0.006)	0.008 (0.006)	0.002 (0.006)	0.004 (0.007)	0.003 (0.006)	−0.005 (0.006)	−0.002 (0.006)
Pooling	−0.005 (0.006)	0.010 (0.006)	0.003 (0.008)	−0.008 (0.009)	0.003 (0.006)	−0.007 (0.010)	−0.009 (0.013)

Table S3: The average posterior SD of the estimated treatment effect in the simulation study based on 500 simulated data sets

Method	Scenario for between-study heterogeneity						
	No	RI+Low	RI+Moderate	RI+High	RIS+Low	RIS+Moderate	RIS+High
$\beta_2 = 0$							
No borrowing	0.142	0.142	0.141	0.142	0.142	0.142	0.141
Conditional MPP	0.123	0.124	0.130	0.134	0.124	0.134	0.137
Marginal MPP	0.128	0.130	0.133	0.136	0.130	0.136	0.138
Commensurate prior	0.138	0.138	0.138	0.139	0.139	0.140	0.140
Pooling	0.123	0.123	0.124	0.126	0.123	0.124	0.126
$\beta_2 = 0.36$							
No borrowing	0.142	0.142	0.142	0.142	0.143	0.142	0.143
Conditional MPP	0.123	0.124	0.131	0.134	0.125	0.134	0.137
Marginal MPP	0.128	0.130	0.133	0.136	0.131	0.136	0.140
Commensurate prior	0.138	0.138	0.139	0.139	0.139	0.140	0.142
Pooling	0.123	0.123	0.124	0.124	0.124	0.125	0.127

Note: All the Monte Carlo errors are less than 0.001 thus not reported in the table.

Table S4: The average MSE (Monte Carlo SE in parentheses) of the estimated treatment effect in the simulation study based on 500 simulated data sets

Method	Scenario for between-study heterogeneity						
	No	RI+Low	RI+Moderate	RI+High	RIS+Low	RIS+Moderate	RIS+High
$\beta_2 = 0$							
No borrowing	0.021 (0.001)	0.021 (0.001)	0.020 (0.001)	0.020 (0.001)	0.020 (0.001)	0.019 (0.001)	0.021 (0.001)
Conditional MPP	0.016 (0.001)	0.018 (0.001)	0.021 (0.001)	0.022 (0.001)	0.018 (0.001)	0.023 (0.001)	0.026 (0.002)
Marginal MPP	0.016 (0.001)	0.017 (0.001)	0.019 (0.001)	0.020 (0.001)	0.017 (0.001)	0.020 (0.001)	0.024 (0.001)
Commensurate prior	0.019 (0.001)	0.019 (0.001)	0.018 (0.001)	0.018 (0.001)	0.019 (0.001)	0.018 (0.001)	0.021 (0.001)
Pooling	0.016 (0.001)	0.018 (0.001)	0.032 (0.002)	0.047 (0.003)	0.019 (0.001)	0.049 (0.003)	0.088 (0.005)
$\beta_2 = 0.36$							
No borrowing	0.020 (0.001)	0.021 (0.002)	0.022 (0.001)	0.022 (0.001)	0.019 (0.001)	0.021 (0.001)	0.019 (0.001)
Conditional MPP	0.016 (0.001)	0.018 (0.001)	0.023 (0.002)	0.024 (0.001)	0.018 (0.001)	0.026 (0.002)	0.023 (0.001)
Marginal MPP	0.016 (0.001)	0.018 (0.001)	0.021 (0.001)	0.022 (0.001)	0.017 (0.001)	0.023 (0.002)	0.021 (0.001)
Commensurate prior	0.019 (0.001)	0.019 (0.001)	0.020 (0.001)	0.020 (0.001)	0.018 (0.001)	0.020 (0.001)	0.018 (0.001)
Pooling	0.016 (0.001)	0.018 (0.001)	0.032 (0.002)	0.044 (0.003)	0.019 (0.001)	0.054 (0.003)	0.085 (0.005)

Distributions of the power parameter in the conditional MPP and the marginal MPP in scenarios without treatment effect are visualized using box plots in the main text. The medians and IQRs of the posterior means of the power parameter for the conditional MPP and the marginal MPP in different scenarios are shown in Table S5, which indicates both methods can incorporate the historical information adaptively.

Table S5: The median (IQR in the parentheses) of the posterior means of the power parameter in the conditional MPP and the marginal MPP in all simulation scenarios based on 500 simulated data sets

Method	Scenario for between-study heterogeneity						
	No	RI+Low	RI+Moderate	RI+High	RIS+Low	RIS+Moderate	RIS+High
$\beta_2 = 0$							
Conditional MPP	0.82 (0.76, 0.85)	0.81 (0.76, 0.85)	0.75 (0.35, 0.82)	0.64 (0.02, 0.81)	0.80 (0.75, 0.84)	0.70 (0.14, 0.80)	0.26 (0.01, 0.77)
Marginal MPP	0.65 (0.60, 0.69)	0.64 (0.57, 0.69)	0.51 (0.26, 0.64)	0.37 (0.13, 0.62)	0.62 (0.54, 0.67)	0.43 (0.23, 0.58)	0.25 (0.09, 0.53)
$\beta_2 = 0.36$							
Conditional MPP	0.82 (0.77, 0.86)	0.81 (0.75, 0.85)	0.75 (0.46, 0.83)	0.65 (0.03, 0.81)	0.81 (0.76, 0.85)	0.70 (0.07, 0.81)	0.28 (0.01, 0.77)
Marginal MPP	0.66 (0.61, 0.70)	0.63 (0.55, 0.69)	0.53 (0.29, 0.64)	0.37 (0.16, 0.60)	0.64 (0.56, 0.69)	0.44 (0.20, 0.60)	0.26 (0.11, 0.50)

5 Sensitivity analysis on the prior for the power parameter

Based on above performance measures of the MPP with Beta(1, 1) prior for the power parameter, the type I error rate can be inflated when the between-study heterogeneity is moderate or high, which implies the current version of MPP is too optimistic in borrowing historical information. Therefore we did a sensitivity analysis using a more skeptical prior, Beta(1, 2), for the power parameter with more probability density near zero to evaluate its effect on the control of type I error rate.

Table S6: The median (IQR in parentheses) of the posterior means of the power parameter in the conditional MPP and the marginal MPP in all simulation scenarios with Beta(1, 2) prior for the power parameter

Method	Scenario for between-study heterogeneity						
	No	RI+Low	RI+Moderate	RI+High	RIS+Low	RIS+Moderate	RIS+High
$\beta_2 = 0$							
Conditional MPP	0.73 (0.68, 0.76)	0.72 (0.67, 0.76)	0.64 (0.12, 0.73)	0.40 (0.02, 0.71)	0.71 (0.65, 0.76)	0.54 (0.06, 0.71)	0.09 (0.01, 0.66)
Marginal MPP	0.51 (0.46, 0.55)	0.50 (0.44, 0.54)	0.40 (0.22, 0.49)	0.29 (0.12, 0.47)	0.48 (0.42, 0.53)	0.34 (0.20, 0.45)	0.21 (0.09, 0.41)
$\beta_2 = 0.36$							
Conditional MPP	0.74 (0.69, 0.77)	0.71 (0.65, 0.76)	0.64 (0.18, 0.74)	0.43 (0.02, 0.71)	0.72 (0.66, 0.77)	0.55 (0.04, 0.71)	0.10 (0.01, 0.63)
Marginal MPP	0.51 (0.47, 0.55)	0.49 (0.42, 0.54)	0.40 (0.25, 0.50)	0.30 (0.14, 0.46)	0.49 (0.43, 0.54)	0.34 (0.18, 0.46)	0.22 (0.10, 0.39)

Table S7: The type I error rate and statistical power (%) of the estimated treatment effect (Monte Carlo SE in parentheses) in the simulation study with Beta(1, 2) prior for the power parameter

Method	Scenario for between-study heterogeneity						
	No	RI+Low	RI+Moderate	RI+High	RIS+Low	RIS+Moderate	RIS+High
$\beta_2 = 0$							
Conditional MPP	5.4 (1.0)	5.6 (1.0)	7.4 (1.2)	8.6 (1.3)	6.0 (1.1)	7.2 (1.2)	9.8 (1.3)
Marginal MPP	5.2 (1.0)	4.8 (1.0)	5.6 (1.0)	6.2 (1.1)	5.0 (1.0)	5.0 (1.0)	8.0 (1.2)
$\beta_2 = 0.36$							
Conditional MPP	81.8 (1.7)	81.6 (1.7)	77.0 (1.9)	74.2 (2.0)	80.2 (1.8)	73.6 (2.0)	71.6 (2.0)
Marginal MPP	80.8 (1.8)	80.2 (1.8)	77.6 (1.9)	76.2 (1.9)	79.8 (1.8)	74.4 (2.0)	71.4 (2.0)

As can be seen from Table S6, estimates of the power parameter are lower than those with a Beta(1, 1) prior due to this more skeptical prior. Although the type I error rate was slightly reduced compared to the original MPP when there was between-study heterogeneity according to Table S7, the Beta(1,2) prior still led to inflated type I error rates in the above scenarios. Hence the prior for the power parameter should be even more skeptical to control the type I error rate.

6 Sensitivity analysis on the LKJ prior in the real data analysis

In the analysis of ADCS data sets, the LKJ(1) prior was used for the correlation matrix in the covariance matrix of random effects. The choice of the hyperparameter η in the LKJ prior may influence the inference for the parameters of interest. A hyperparameter $\eta < 1$ favors more correlation while $\eta > 1$ favors less correlation. Therefore, we conducted a sensitivity analysis on the choice of η ($\eta = 0.5, 2$) using the ADCS data. The results are shown in Figure S8, which are almost identical to those based on the LKJ(1) prior.

Table S8: Parameter estimates of the ADC-027 trial using different borrowing methods with different LKJ priors

η	Method	Time effect β_5			Treatment effect β_6		
		Posterior mean	Posterior SD	95% CI	Posterior mean	Posterior SD	95% CI
0.5	No borrowing	0.521	0.039	(0.445, 0.599)	−0.022	0.051	(−0.124, 0.078)
	Conditional MPP	0.462	0.026	(0.412, 0.514)	0.033	0.040	(−0.044, 0.112)
	Marginal MPP	0.477	0.032	(0.415, 0.541)	0.021	0.045	(−0.069, 0.109)
	Commensurate prior	0.514	0.039	(0.440, 0.590)	−0.015	0.051	(−0.115, 0.083)
	Pooling	0.452	0.024	(0.404, 0.501)	0.041	0.040	(−0.036, 0.118)
2	No borrowing	0.521	0.039	(0.444, 0.599)	−0.022	0.051	(−0.124, 0.079)
	Conditional MPP	0.461	0.026	(0.412, 0.511)	0.035	0.040	(−0.044, 0.113)
	Marginal MPP	0.477	0.032	(0.416, 0.540)	0.022	0.044	(−0.067, 0.108)
	Commensurate prior	0.516	0.039	(0.440, 0.594)	−0.019	0.051	(−0.121, 0.082)
	Pooling	0.452	0.025	(0.403, 0.500)	0.041	0.040	(−0.038, 0.121)

The medians and the IQRs of the power parameter are 0.63 (0.56, 0.73) and 0.70 (0.61, 0.78) for the conditional MPP with $\eta = 0.5, 2$, respectively. For the marginal MPP, the medians and IQRs of the power parameter are 0.41 (0.32, 0.54) and 0.42 (0.32, 0.56), respectively.

7 Graphical convergence diagnostics for the motivating data analysis

In this section, we present the trace plots and the autocorrelation plots for parameters of interest of the methods involved in Section 7 of the paper. The plots have shown that all the methods have achieved convergence in the real data analysis.

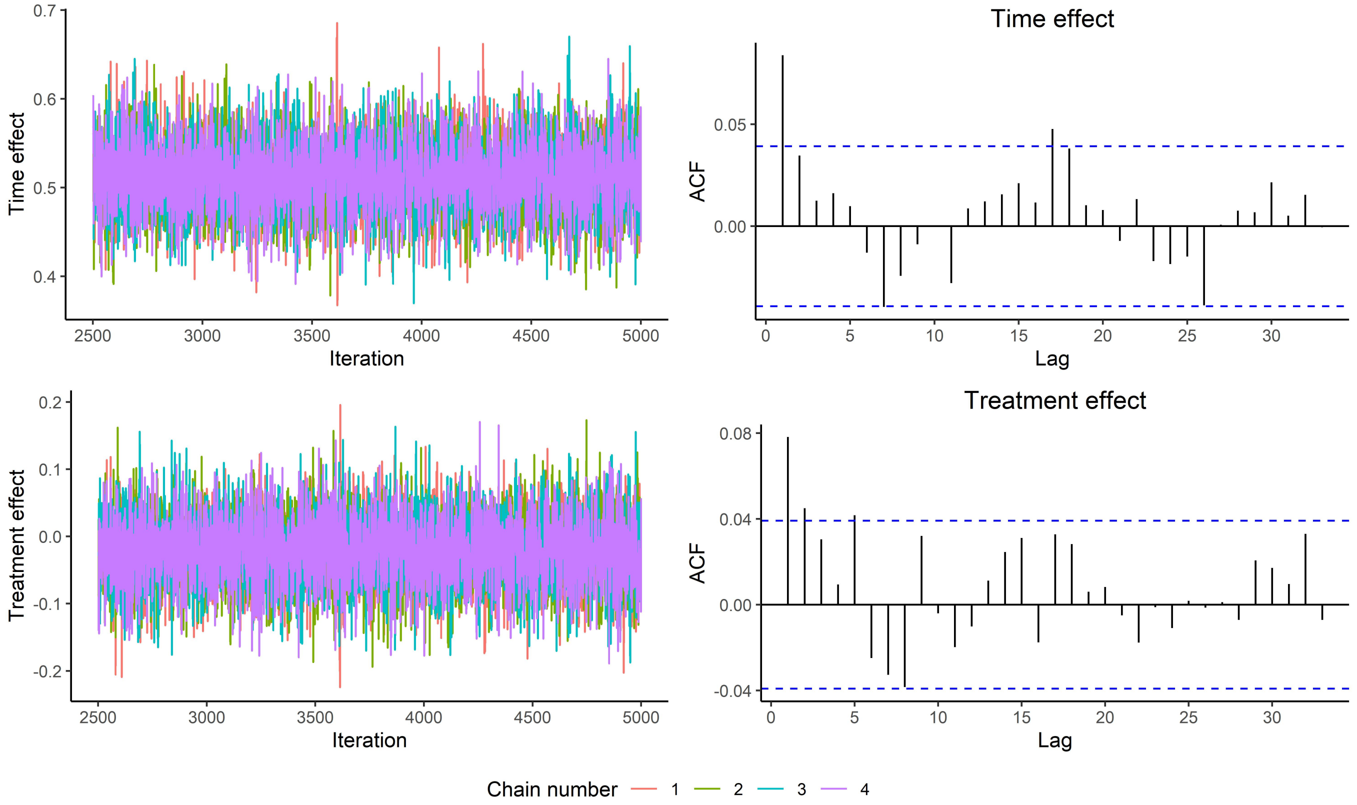

Figure S1: Trace plots and autocorrelation plots for the time effect and the treatment effect in the no borrowing method

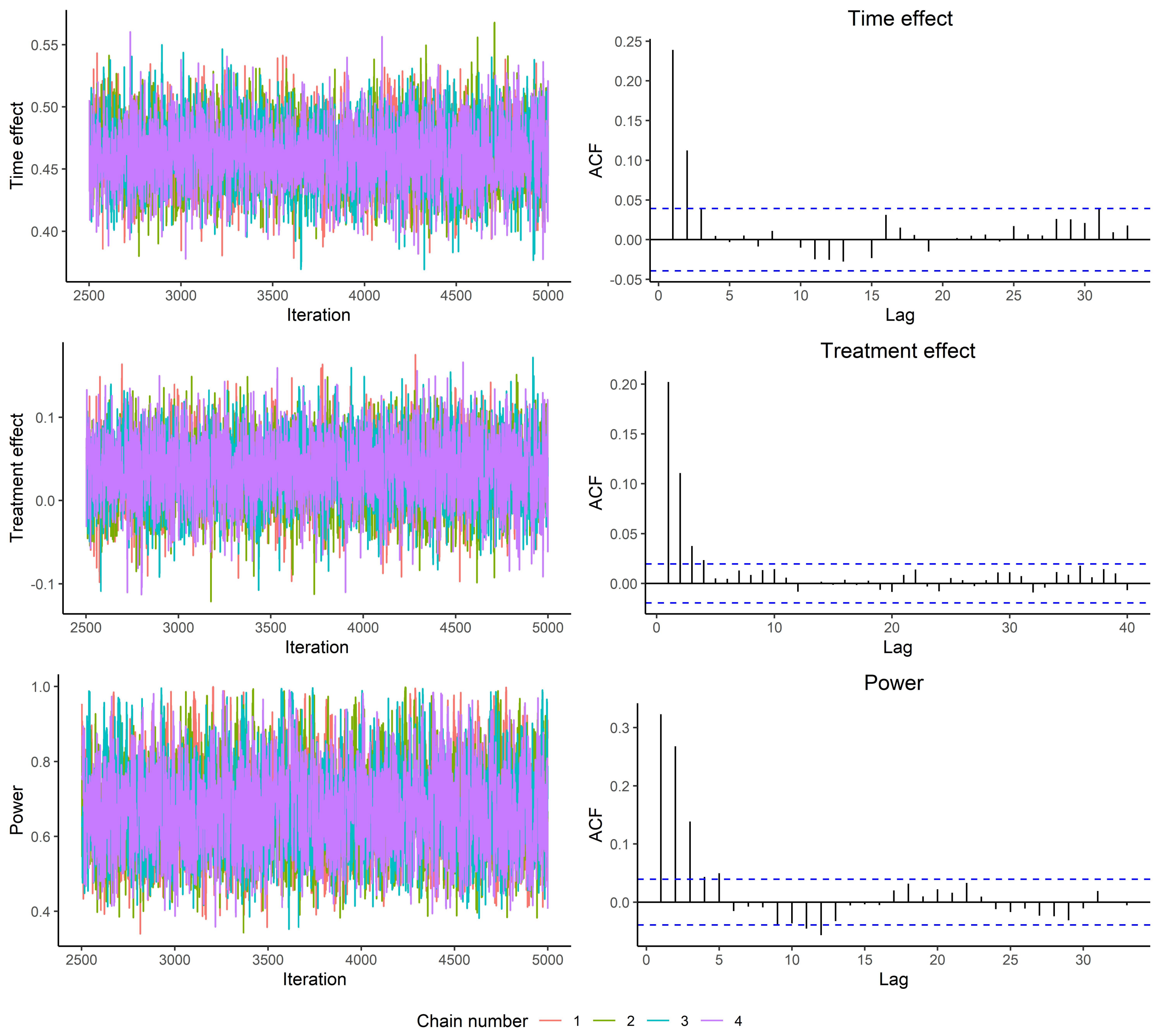

Figure S2: Trace plots and autocorrelation plots for the time effect, the treatment effect and the power parameter in the conditional MPP method

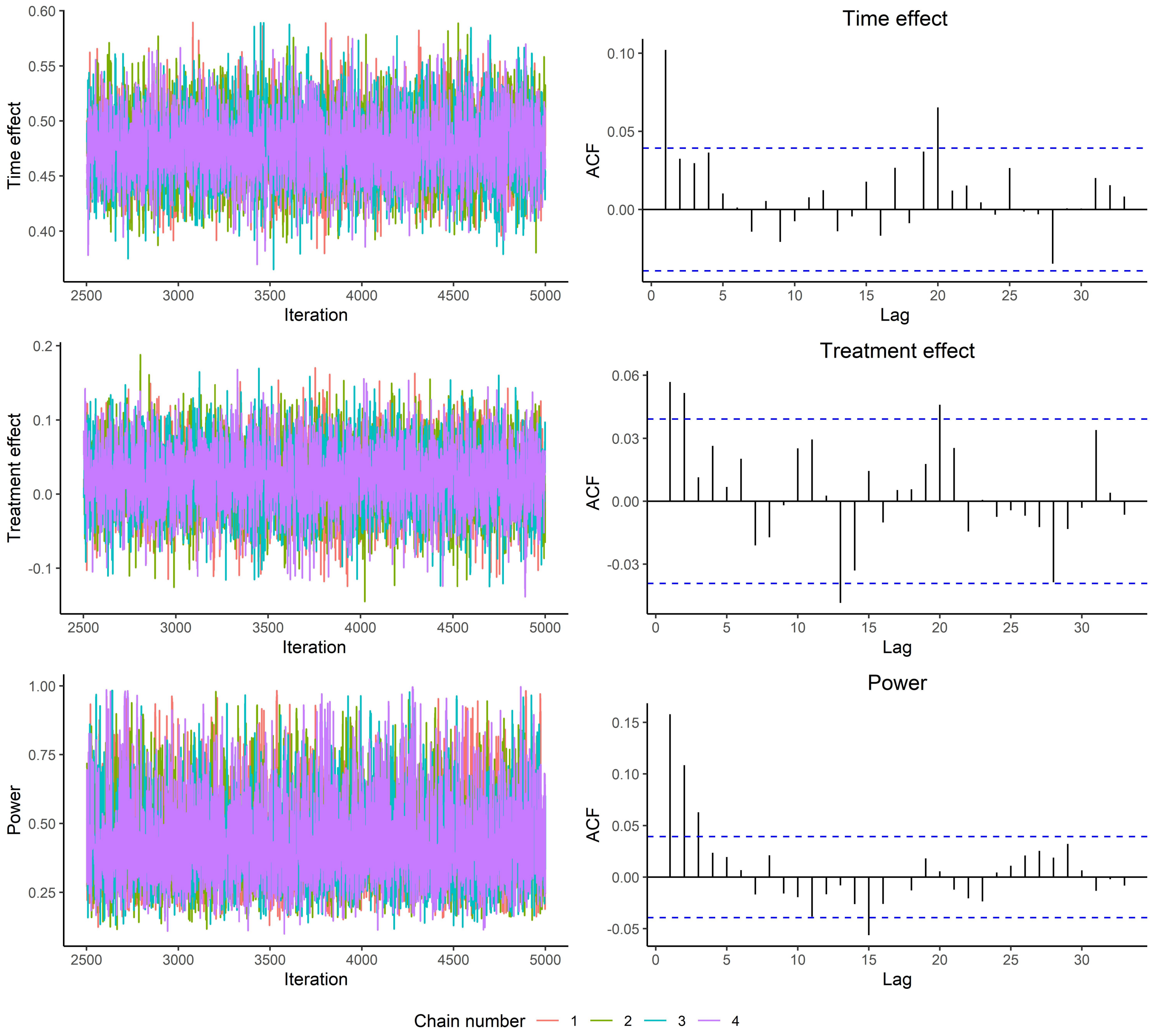

Figure S3: Trace plots and autocorrelation plots for the time effect, the treatment effect and the power parameter in the marginal MPP method

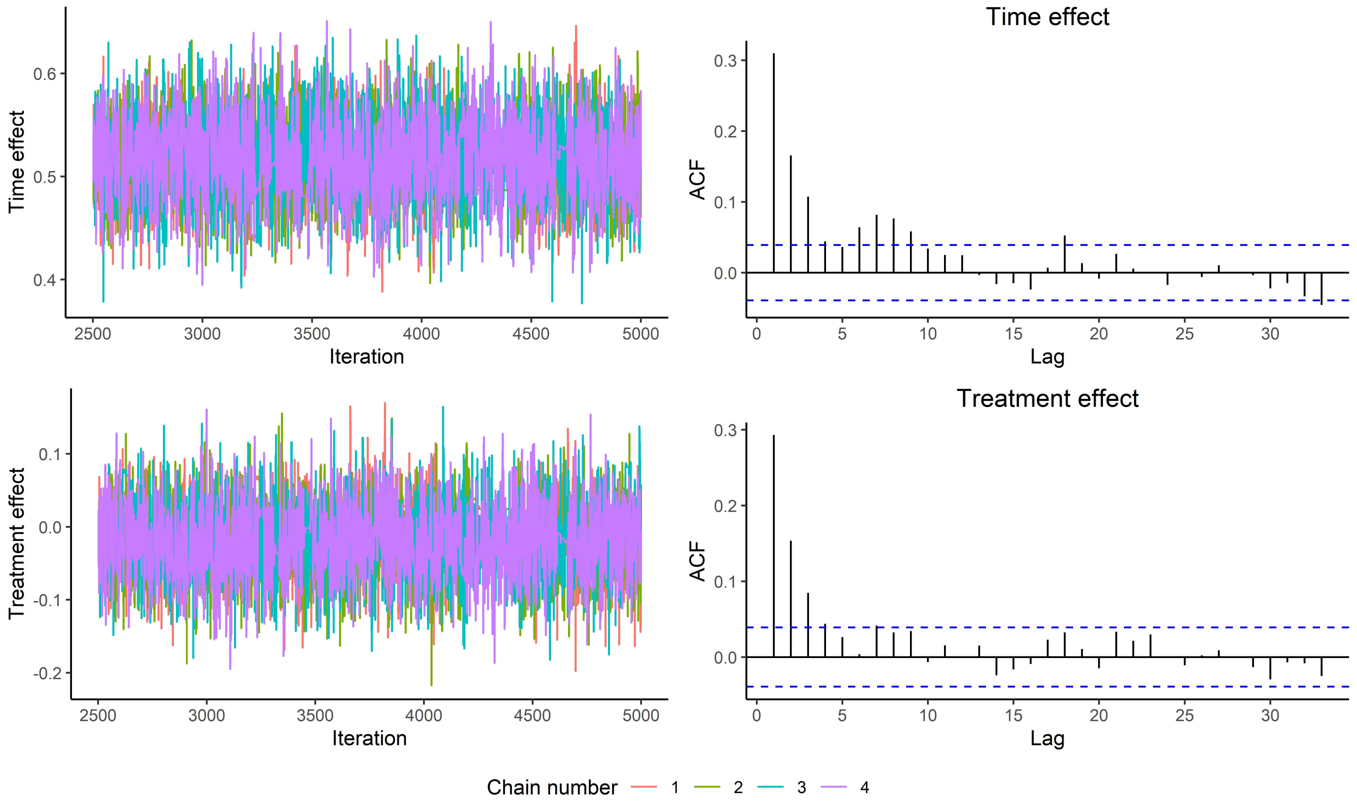

Figure S4: Trace plots and autocorrelation plots for the time effect and the treatment effect in the commensurate prior method

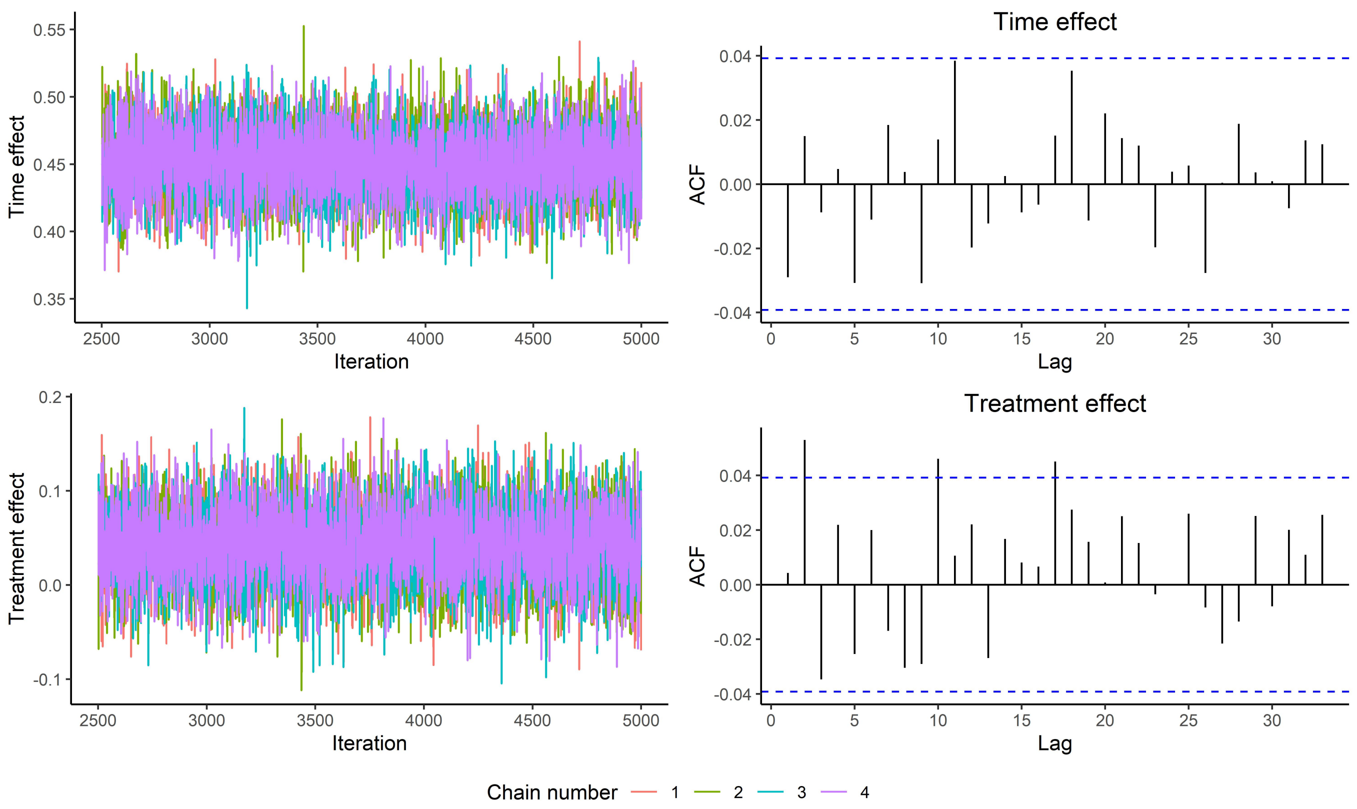

Figure S5: Trace plots and autocorrelation plots for the time effect and the treatment effect in the pooling method

8 Source code

The R and Stan syntax files for the methods in the simulation study are available on a GitHub repository (`https://github.com/QiHongchao/MPP_longitudinal`).

References

[1] Joost van Rosmalen, David Dejardin, Yvette van Norden, Bob Löwenberg, and Emmanuel Lesaffre. Including historical data in the analysis of clinical trials: Is it worth the effort? *Stat Methods Med Res.*, 27(10):3167–3182, 2018.
